# Prediction of protein interaction types based on sequence and network features

**DOI:** 10.1186/1752-0509-7-S6-S5

**Published:** 2013-12-13

**Authors:** Florian Goebels, Dmitrij Frishman

**Affiliations:** 1 Department of Genome Oriented Bioinformatics, Wissenschaftszentrum Weihenstephan, Maximus-von-Imhof-Forum 1, Freising, 85350, Germany; 2 HMGU German Research Center for Environmental Health, Institute for Bioinformatics and Systems Biology/MIPS, Ingolstadter Landstraße 1, Neuherberg, 85764, Germany

**Keywords:** protein-protein interactions, biological network analysis, protein structure prediction, systems biology, sequence analysis

## Abstract

**Background:**

Protein interactions mediate a wide spectrum of functions in various cellular contexts. Functional versatility of protein complexes is due to a broad range of structural adaptations that determine their binding affinity, the number of interaction sites, and the lifetime. In terms of stability it has become customary to distinguish between obligate and non-obligate interactions dependent on whether or not the protomers can exist independently. In terms of spatio-temporal control protein interactions can be either simultaneously possible (SP) or mutually exclusive (ME). In the former case a network hub interacts with several proteins at the same time, offering each of them a separate interface, while in the latter case the hub interacts with its partners one at a time via the same binding site. So far different types of interactions were distinguished based on the properties of the corresponding binding interfaces derived from known three-dimensional structures of protein complexes.

**Results:**

Here we present PiType, an accurate 3D structure-independent computational method for classifying protein interactions into simultaneously possible (SP) and mutually exclusive (ME) as well as into obligate and non-obligate. Our classifier exploits features of the binding partners predicted from amino acid sequence, their functional similarity, and network topology. We find that the constituents of non-obligate complexes possess a higher degree of structural disorder, more short linear motifs, and lower functional similarity compared to obligate interaction partners while SP and ME interactions are characterized by significant differences in network topology. Each interaction type is associated with a distinct set of biological functions. Moreover, interactions within multi-protein complexes tend to be enriched in one type of interactions.

**Conclusion:**

PiType does not rely on atomic structures and is thus suitable for characterizing proteome-wide interaction datasets. It can also be used to identify sub-modules within protein complexes. PiType is available for download as a self-installing package from http://webclu.bio.wzw.tum.de/PiType/PiType.zip.

## Background

Detailed protein interaction maps derived for many important model organisms [1] have become one of the principal tools of systems biology research. A wide range of high-throughput experimental methods is available today for detecting protein interactions at proteome scale, but they essentially provide a binary readout - whether or not two proteins form a complex - and give no clue as to how strongly the protomers interact with each other, how long the interaction lasts, and in which order multiple interaction partners associate with each other. Knowledge about the lifetime and binding affinity of non-covalent protein assemblies is crucial for understanding their mode of action and their role in cellular processes.

So far most of the mechanistic insights into the nature of protein interactions came from high-resolution structures of protein complexes [2,3]. One important distinction can be made between obligate and non-obligate interactions, dependent on whether or not the protomers can exist independently from each other. The interfaces of non-obligate interactions tend to be smaller, less tightly packed, more polar, less conserved, and overall more similar to normal protein surfaces in terms of amino acid composition than those of obligate interactions [4-9].

Protein complexes can also be subdivided into two classes based on their binding affinity and lifetime. Constituents of permanent interactions, such as enzyme-inhibitor or antibody-antigen complexes, are only found in bound state while transient interactions, usually involved in intracellular signaling, are short-lived and readily associate and dissociate [2]. Interaction sites of transient protein complexes have the tendency to be disordered and their binding specificity is often determined by short linear amino acid motifs (ELM) [3,10]. Obligate interactions are usually permanent [2] whereas non-obligate interactions are mostly transient [11].

Several machine learning methods have been proposed to automatically classify protein complexes with known three-dimensional structure into various types based on physical, chemical, geometrical, and evolutionary properties of protein recognition sites [12-20]. For example, Mintseris and Weng achieved an accuracy of 91% in separating transient from permanent complexes using atomic contact vectors to describe the properties of interaction interfaces [20]. Likewise, the NOXclass classifier developed by Zhu et al [17] distinguishes obligate from non-obligate interactions with an accuracy of 91.8% by considering the interface area, amino acid composition, shape complementarity, and evolutionary conservation.

Protein interactions can also be classified into two types based on their timing and the spatial distribution of binding sites on the protein surface. Products of co-expressed genes [21] may form stable complexes and interact with each other simultaneously, which is only possible when a network hub ("party hub") possesses a unique binding site for each interaction partner [22]. Alternatively, hub proteins that are not co-expressed with their interaction partners are believed to bind their partners individually at different times (or in different cellular locations) via the same interface ("date hubs") [22]. Following Kim et al. [22] we refer to the interactions of the first and the second type as simultaneously possible (SP) and mutually exclusive (ME), respectively. SP and ME interactions and the corresponding binding interfaces can be directly studied by overlaying high-quality protein interaction data with known three-dimensional structures of protein complexes. Analyses of such a structurally resolved interaction network (SIN) together with gene expression patterns revealed distinctly different cellular roles of party and date hubs, with the former corresponding to stable network modules and the latter connecting modules with each other. Date hubs show much lower average degree and are more often encoded by essential genes than party hubs. As well, proteins involved in SP interactions (and hence co-expressed) tend to be more functionally similar than those involved in ME interactions, which led to the suggestion that ME interactions are mostly transient [22] while SP interactions are preferentially obligate [23].

So far efforts to classify and predict protein interaction types have exploited structural information and are thus only applicable to the minor part of the currently known interactome for which atomic structures of protein complexes are available. Here we present the first attempt to classify protein interactions without reliance on 3D structures. We have devised an accurate prediction technique, called PiType, which is able to distinguish obligate from non-obligate interactions and SP from ME interactions based on readily accessible features, including sequence and functional properties of the two binding partners and their network context. We apply PiType to large-scale protein interaction data and investigate the cross-talk between SP/ME and obligate/non-obligate interactions.

## Materials and methods

### Protein sequences, structures, and annotations

Protein sequences and associated annotation for *Homo sapiens, Escherichia coli*, and *Saccharomyces cerevisiae *were extracted from the Uniprot database [24] based on the taxon identifiers of these organisms (9606, 83333, and 559292, respectively). We only considered manually reviewed Uniprot entries to reduce the influence of wrong gene models on our results. If a protein had several annotated isoforms we selected the longest one.

To establish the correspondence between known three-dimensional structures and the protein sequences in our dataset we used both the Uniprot-to-PDB mapping available from the Uniprot ftp site and the PDB-to-Uniprot mapping available through the PDB [25] SOAP service. The Uniprot-to-PDB mapping was reversed (*i.e*. converted to a list of PDB IDs corresponding to Uniprot IDs) and then merged with the PDB-to-Uniprot mapping. All PDB chain IDs that corresponded to more than one Uniprot ID were removed, but we allowed an Uniprot ID to be mapped to several different PDB chain IDs.

Gene ontology [26] assignments were obtained through the QuickGO [24] proteome download page based on taxonomic identifiers. Summary statistics about protein information used in this work are shown in Table 1.

**Table 1:** Overview of the protein information used in this work.

	Human	Yeast	*E. coli*
Reviewed proteins in Uniprot	20226	6619	4303

PDB chain IDs mapped to reviewed Uniprot entries	41605	7585	15661

Proteins with at least one mapped PDB chain ID	4519	920	1223

Proteins with at least one GO annotation	18283	5908	3744

### Dataset of obligate and non-obligate interactions

There are two well-known manually curated datasets of protein interaction types created by Zhu et al. [17] and Mintseris et al. [9,20]. In these datasets a non-redundant set of protein complexes with known three-dimensional structure from 80 different species was classified into obligate and non-obligate (which also includes transient). However, the Mintseris dataset is not directly suitable for training our classifier as it distinguishes transient non-obligate and permanent obligate protein interactions, neglecting permanent non-obligate interactions; we do use this set for classifier evaluation (see Additional file 1). The Zhu dataset was created by combining two data sources: i) a non-redundant set of protein complexes from the PDB database for which literature evidence indicates that they occur naturally and are stable as a dimer [18], and ii) a set of non-obligate interactions corresponding to protein pairs that are found in the PDB database both in the bound and unbound state [27]. In total this dataset contains 137 interactions and was used to evaluate several structure-based classifiers of protein interaction types [13-15]; however, it contains only 25 data points for human, yeast, and *E. coli *and is hence insufficient for our study.

We therefore created a larger dataset by predicting the interaction type of *E. coli*, yeast and human complexes by a structure based classifier, NOXclass [17]. NOXclass employs a two-stage support vector machine (SVM) algorithm to first filter out crystal artifacts and then to classify complex structures as obligate and non-obligate. The NOXclass SVM was reported to achieve the highest classification accuracy (90.9%) using the following structural features: interface area, interface area ratio, area based amino acid composition, and gap volume index. For calculating the former three features NOXclass requires the NACCESS tool [28] while the latter feature is computed using SURfnet [29].

We generated a dataset of obligate and non-obligate interactions with the NOXclass predictor. A list of all structures from human, yeast or E coli with at least two chains in the biological unit was retrieved from the PDB database. Protein chains that could not be mapped to Uniprot entries (see above) were ignored. If a PDB entry contained more than two chains we considered all possible chain combinations and classified them using NOXclass. Two confidence values were obtained for each pair of protein chains - one for classifying this chain pair as a biological assembly or a crystal artifact, and another one for obligate *vs *non-obligate complexes. To generate our dataset we accepted only those protein chains for which NOXclass produced confidence values of at least 90% at both stages. The NOXclass predictions were subsequently merged with the manually annotated interactions from the Zhu dataset. In total we obtained 773 protein protein interactions with known or reliably predicted interaction type (Table 2).

**Table 2:** Sizes of the datasets of obligate and non-obligate interactions for different organisms.

Organism	Obligate interactions	Non-obligate interactions
Human	121	423

Yeast	115	55

*E. coli*	45	15

Total	280	493

### Structural interaction network

The Structural Interaction Network 2.0 [30] (SIN) combines structurally resolved protein complexes into a comprehensive protein interaction network. The database was generated by first selecting experimentally determined high-confidence interactions in human and yeast from the BioGrid [31] database. Each interaction is then mapped to available PDB structures by sequence similarity. A unique feature of this resource is the classification of interactions into mutually exclusive and simultaneously possible ones. A protein interaction is said to be mutually exclusive, if two or more proteins interact with the same interface on the surface of their common partner. Otherwise, if the interactors bind at different sites of their common partner, the interaction is considered simultaneously possible. We obtained information from SIN on 3096 mutually exclusive and 816 simultaneously possible interactions in human as well as on 584 mutually exclusive and 117 simultaneously possible interactions in yeast.

### Protein interaction data

Protein interaction data for yeast, human, and *Escherichia coli *were obtained from the iRefIndex 9.0 meta-database [32] which stores and cross-references information from various resources, including DIP, MINT, Intact, Biogrid, and HPRD [31,33-36]. We considered only information on direct physical interactions measured by a variety of methods such as yeast two hybrid, tandem affinity, anti tag/bait coimmunoprecipitation, etc. An overview of the network size and the experimental data is given in Table 3.

**Table 3:** Sizes of protein interaction networks in human, yeast, and *E. coli*.

	Human	Yeast	*E. coli*	Total
Nodes	9917	5528	2068	17513

Non-redundant interactions	41115	39045	7197	87357

Raw interactions	77742	59336	13068	150146

Yeast two hybrid (MI:0018)	13876	11055	54	24985

Anti-tag coimmunoprecipitation (MI:0007)	1311	10049	0	11360

Pull down (MI:0096)	6651	4024	78	10753

Experimental interaction detection (MI:0045)	8243	23	6	8272

Enzymatic study (MI:0415)	1002	2	4	6491

Inferred by author (MI:0363)	0	388	5898	6286

Anti-bait coimmunoprecipitation (MI:0006)	5984	22	2	6008

Tandem-affinity purification (MI:0676)	317	3631	1112	5060

Others	40358	30142	5914	76414

Non-redundant interactions: each unique combination of interactors A and B is counted as a single interaction, regardless of directionality, experimental system, and data source. Raw interactions: Each unique combination of interactors A and B, experimental system and data source is counted as a single interaction^a^. Each interaction detection method is annotated with its Ontology Term (e.g. MI:0018).

### Protein features used for machine learning

#### Edge graphlet degree vectors

**Figure 1:**
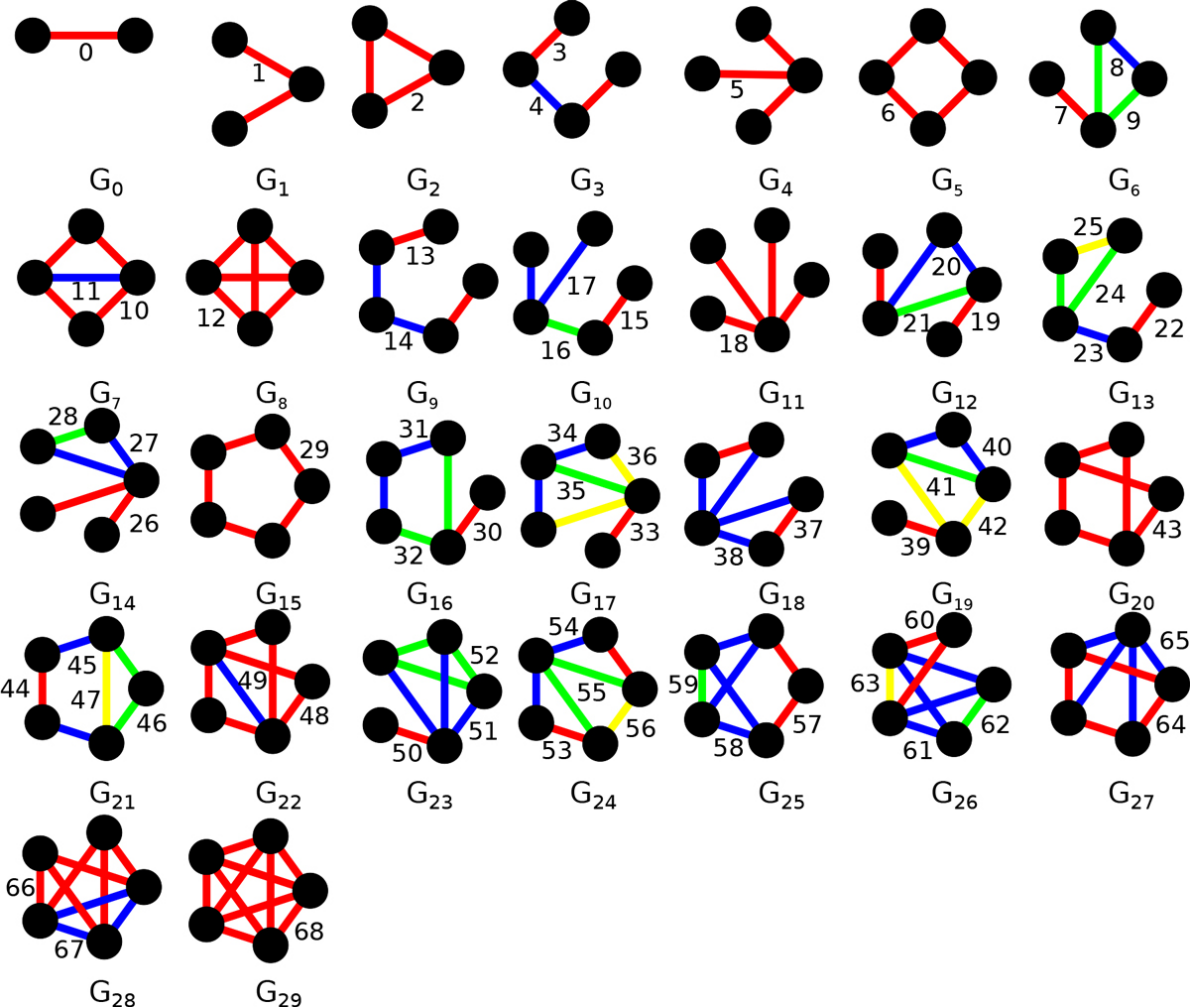
**All possible graphlets of size 2 to 5 containing all 69 topologically unique edge orbits**. Each unique edge orbit inside each graphlet is marked with a different color. For example, in the graphlet G_13 _edge orbits 22, 23, 24, and 25 are colored red, blue, green, and yellow, respectively.

**Figure 2:**
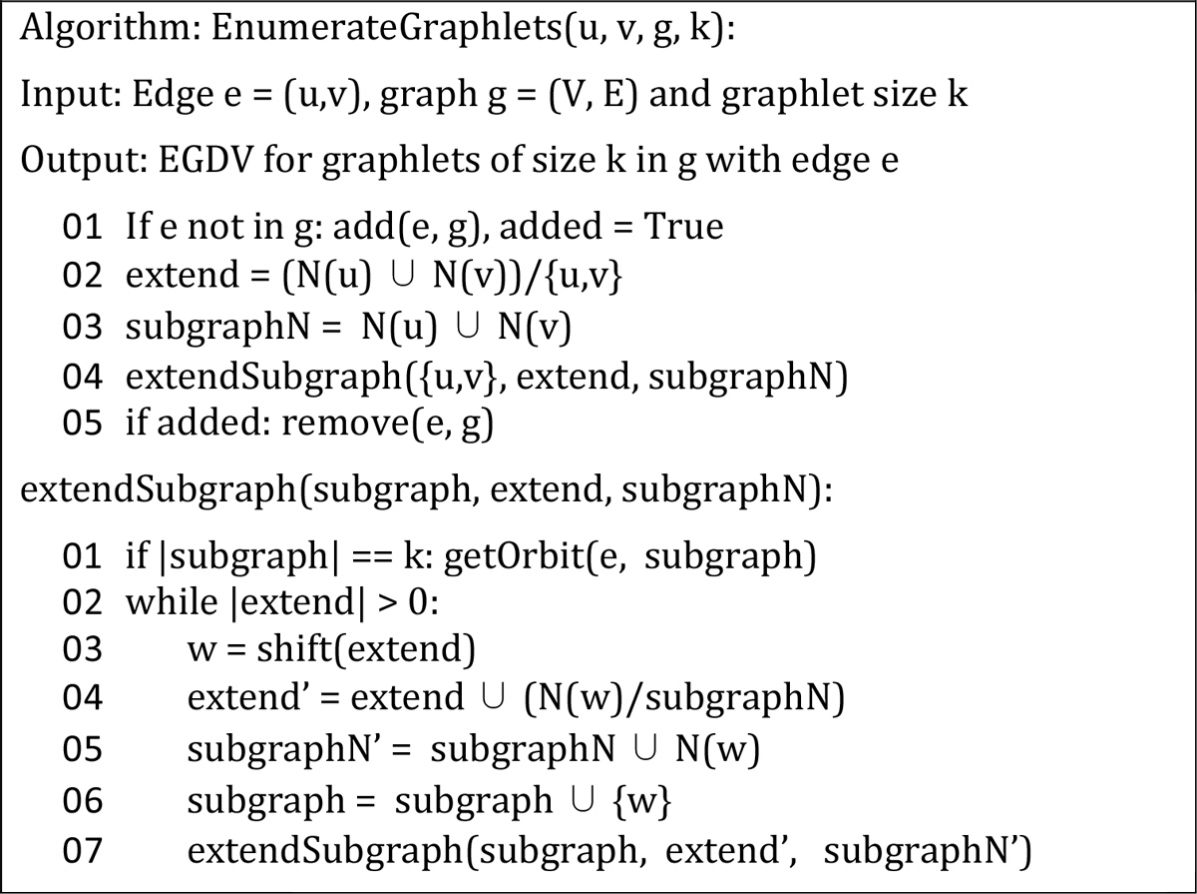
**Pseudo code for the EGGV calculation algorithm**. N(v) denotes the neighborhood of v, *i.e*. all nodes that share an edge with v. A/B denotes subtraction, for example: {1,2,3,5}/{2,3} = {1,5}. A∩B denotes a union of two sets, for example: {1,2}∪{2,3} = {1,2,3}.

We used edge graphlet degree vectors (EGDV) [37] as a method for measuring the local topology of an edge *e *in a graph *g*. Graphlets are small, connected, induced subgraphs of a larger network (Figure 1). In this work we consider graphlets of size two to five (*i.e*. having between two and five nodes). The local topology of an edge *e *can be determined by counting how often *e *is contained in all graphlets of size two to five in *g*. Moreover, one has to differentiate at which position *e *resides in a graphlet. For example, there are two distal edges at both ends of the graphlet G_3 _and as well as one edge in the middle. To distinguish between such cases the symmetry of each edge is described by its atomorphism orbits. There is a total of 69 orbits, numbered 0 to 68. However, the orbit 0 consists of just one edge connecting two nodes. Since each edge in *g *touches this orbit exactly once (namely itself), it is not considered while calculating EGDV. We used a modified version of the FANMOD algorithm [38] to find all graphlets in g which contain a specific edge *e *(Figure 2) and determined the orbit of *e *in the graphlet using the *nauty *package [39]. Since values of the EGDV tend to be very large and are difficult to compare we transformed them to the natural logarithmic scale and normalized them by dividing each value by the total sum of all orbits in the EGDV (thus the sum of each orbit is 1).

We sought to identify the preferred network contexts for protein interactions of different types. To this end we investigated the enrichment of orbits in two specific local topological patterns - clusters and hubs. Edges constituting a highly connected sub-graph (cluster) would be expected to be enriched in orbits situated inside cliques, such as 2, 12, 8, 25, 52, and 68 (Figure 1). To be more specific orbits 2, 8, 25, 52 lie within the 3-node clique (G_2_), orbit 12 within the 4-node clique (G_8_), and orbit 68 within the 5-node clique (G_29_). This over-representation of certain orbits is the consequence of the large amount of combinatorial occurrences of different graphlets in tightly connected network clusters. For example, a fully connected 10-node clique, in which each node is connected to each other node, will contain the 3, 4, and 5 node sub-cliques exactly $\left(\begin{array}{c} 10\\ 3 \end{array}\right)$, $\left(\begin{array}{c} 10\\ 4 \end{array}\right)$, and $\left(\begin{array}{c} 10\\ 5 \end{array}\right)$, or 120, 210, and 252, times, respectively. Thus every edge in the 10-node clique touches orbits 2, 12, and 68 exactly $\left(\begin{array}{c} 10\\ 1 \end{array}\right)$, $\left(\begin{array}{c} 10\\ 2 \end{array}\right)$, and $\left(\begin{array}{c} 10\\ 3 \end{array}\right)$, or 10, 45, 120 times, respectively, and every other orbit 0 times. The lower numbers in the binomial coefficients describing orbit counts are two less in comparison to sub-cliques because for every edge the two nodes which it connects are fixed.

Clusters are also enriched in orbits (namely 8, 9, 20, 21, 24, 25, 27, 28, 51, 52, 61, 62, 63) that lie within cliques even if the associated graphlet includes further orbits that do not belong to any clique. For example, the graphlet G_6 _includes three orbits - one 8 orbit and two 9 orbits - that form a 3 node clique as well as the orbit 7 which is a single attached edge to the 3 node clique. For illustration let us now consider a network consisting of 11 nodes, of which 10 nodes form a tightly connected clique, as above. In other words, an additional edge is added to the 10-node clique connecting one of the clique nodes to a node outside of the clique. In all occurrences of G_6 _in this network the newly added edge will correspond to the orbit 7 of G_6 _while the two other orbits of G_6 _, 8 and 9, will lie within the 10 node clique. As a result, orbits 8 and 9 will be enriched for edges belonging to highly connected clusters.

Edges connecting hub nodes and non-hub nodes, as well as those connecting two different hub nodes, are primarily associated with orbits 1, 5, and 18. The reason for this is that hubs tend to have a high degree and a low cluster coefficient, *i.e*. their neighbors are sparsely connected. Edges incident to a hub are thus unlikely to form cliques.

Finally, another crucially important type of network nodes are bottlenecks, which come in two flavors: hub-bottlenecks and nonhub-bottlenecks [40]. Hub-bottlenecks are proteins characterized by high betweeness and high degree; they are situated between protein clusters, such that a large number of the shortest paths pass through them. Nonhub-bottlenecks also display high betweeness, but their degree is low; they are the members of each respective protein cluster which interact with the hub-bottleneck node. Thus, orbits that touch a clique (such as 7, 45, 50, 57, and 58) will be enriched in interactions connecting hub-bottlenecks and nonhub-bottlenecks.

#### PageRank Affinity

We calculated the PageRank Affinity score [41]http://gaussian.bu.edu/pnns.html that describes the closeness of two nodes on the network. It was developed to determine whether or not two nodes share the same graph cluster. Since we expect that obligate interactions will tend to share a cluster the PageRank affinity score may be instrumental in separating non-obligate interactions from the obligate ones.

#### Betweenness

Betweenness (or centrality) of an edge is defined by the number of shortest paths passing through that edge. Since non-obligate interactions are typically involved in signal transduction pathways they would be expected to reside on shortest paths more frequently than obligate interactions. We used the igraph R package [42]http://cran.r-project.org/web/packages/igraph/index.html to calculate edge betweenness.

#### Degree

The degree of an edge *e *is the number of edges that share at least one node with *e*. In other words the degree of an edge between node v_1 _and node v_2 _is the number of edges that have at least v_1 _or v_2 _as a node.

#### Eukaryotic linear motifs (ELM)

It has been suggested that interactions in eukaryotic organisms that are mediated by short linear sequence motifs tend to be non-obligate [3]. To determine the number of ELMs for each protein we downloaded the ELM database [43]http://elm.eu.org/infos/news.html and searched in each protein sequence for all occurrences of each ELM. Hence each interaction was characterized by two integer values giving the numbers of ELMs found in both interaction partners.

#### Disordered binding regions

We predicted disordered binding regions for interacting protein pairs by ANCHOR [44] and considered as features the total number of disordered binding regions, the fraction of disordered amino acids, as well as the length of the longest disordered binding region in both interacting proteins. Hence, for each pair we obtained two values for the number of disordered binding regions and the fractions of disordered amino acids and one value for the length of the longest disordered binding region.

#### Functional similarity

Functional similarity between two proteins was calculated based on their associated Gene Ontology (GO) annotation [45] using the method of Wang et al. [46] as implemented in the GOSemSim package [47]http://www.bioconductor.org/packages/2.4/bioc/html/GOSemSim.html. This method describes the similarity between two GO terms based on their location in the GO graph. To calculate the functional similarity between a protein A having GO terms GO_1_, ..., GO_i _and a protein B with GO_1_, ..., GO_j _all *i *GO terms of A are compared with all *j *GO terms of B, yielding a matrix *m *with *i *rows *j *columns corresponding to GO terms of A and B, respectively. Functional similarity between A and B is then the mean over the maxima of each row and column of *m*:

$$funsim\left(A,B\right)=\frac{\sum_{k=1}^{i}\text{max}\left(m_{i,1..j}\right)+\sum_{l=1}^{j}\text{max}\left(m_{1..i,j}\right)}{i+j}$$

### Machine learning methods

In this section we describe the applied machine learning methods (see Additional file 2 for a more detailed description of machine learning procedures). We used the Weka package[48] (http://www.cs.waikato.ac.nz/ml/weka, v. 3.6.6) and its java API for feature selection and classification.

#### Random forest

Block et al. reported that from all tested classifiers the decision tree method achieved the best performance in distinguishing between permanent and transient interactions based on known three-dimensional structures of protein complexes [16]. Moreover, the random forest algorithm has a better accuracy and is more robust than the decision tree approach [49]. We used the random forest classification algorithm with an ensemble of 10 decision trees. These trees are used to create a confidence value for each predicted class *c*, which lies between 0 and 1. This value describes the fraction of decision trees that voted for class *c*.

### Biological validation

#### Functional enrichment analysis

The goal of the enrichment analysis is to determine whether proteins of the same class share the same molecular function, as defined by Gene Ontology [50-53], more frequently than random proteins. In order to apply this approach to protein interactions the following two circumstances need to be taken into account: a) protein interactions can be both SP and obligate at the same time, since the SP/ME and obligate/non-obligate classifications are independent from each other. Thus all possible combinations of the interaction types (obligate and SP, obligate and ME, non-obligate and SP, non-obligate and ME) need to be analyzed, and b) GO annotation is only available for individual proteins and not for protein interactions. We therefore annotated protein interactions by combining the GO annotation of the two interacting proteins. For an interaction *e *between two proteins A and B we first retrieved all associated GO terms for protein A and protein B, and then annotated *e *only with those GO terms occurring in both protein annotations. The Ontologizer tool [54] was employed to find differences in GO term enrichment between a study set and the general population of protein interactions. P-values were calculated using the Parent-Child-Union method [55]. We conducted a GO enrichment analysis for all four possible class combinations such that the population set and P-value calculation stayed the same while the study set contained interactions with the same predicted class combinations. The Ontologizer represents the enriched GO terms as a hierarchical tree.

#### Protein complex data

In this study we utilized two datasets of multi-protein complexes. One of them was the manually curated collection of 1845 human protein complexes obtained from the CORUM database [56]. Information about pairwise interactions between complex members was extracted from the iRefIndex database (see Table 3). We considered only those 921 CORUM complexes that formed a connected sub-graph in the iRefIndex interaction network, such that there exist a path between any two members of the complex. Furthermore we removed all protein complexes with less than 4 members, leaving us with only 244 complexes.

We also utilized the recently published study of the human protein interaction network, which revealed 13993 high-confidence interactions between 3006 proteins in HeLa S3 and HEK293 cells (further referred to as the HeLa dataset) [57]. These interactions were generated by biochemical fractionation combined with quantitative tandem affinity mass spectrometry and were further stringently filtered by an integrative computational approach, taking into account additional supporting evidence. The authors applied the ClusterOne algorithm [58] to derive 622 putative protein complexes from this network, of which 187 had already been annotated in pubic databases. Note that by design all HeLa complexes form connected sub-graphs. After the publication four proteins were removed from this dataset, reducing the total number of proteins, pairwise interactions, and clusters to 3002, 13979, and 621, respectively. We excluded from consideration 151 protein complexes with less than four members to obtain 470 HeLa protein complexes, of which 163 were previously annotated and 307 were putative, computationally derived complexes.

#### Enrichment of interaction types in protein complexes

We were interested to find out whether network clusters corresponding to protein complexes are enriched in a certain interaction type (SP/obligate, etc.) or are rather a mixture of different interaction types. Such enrichment was assessed based on the information content of a protein complex *c *calculated as

$$R\left(c\right)=\log_{2}4-H\left(c\right)$$

where *H(c) *denotes the Shannon entropy

$$H\left(c\right)=-\overset{\left\{type\right\}}{\underset{t}{\sum }}P\left(t,c\right)*\log_{2}P\left(t,c\right)$$

and P(t, c) the frequency of interaction type t in the protein complex. The information content value ranges between zero and two bits, where a value of two means that all interactions in the protein complex are of the same type and a value of zero indicates that each protein interaction type is equally represented in the protein complex.

## Results and discussion

### Feature analysis

**Figure 3:**
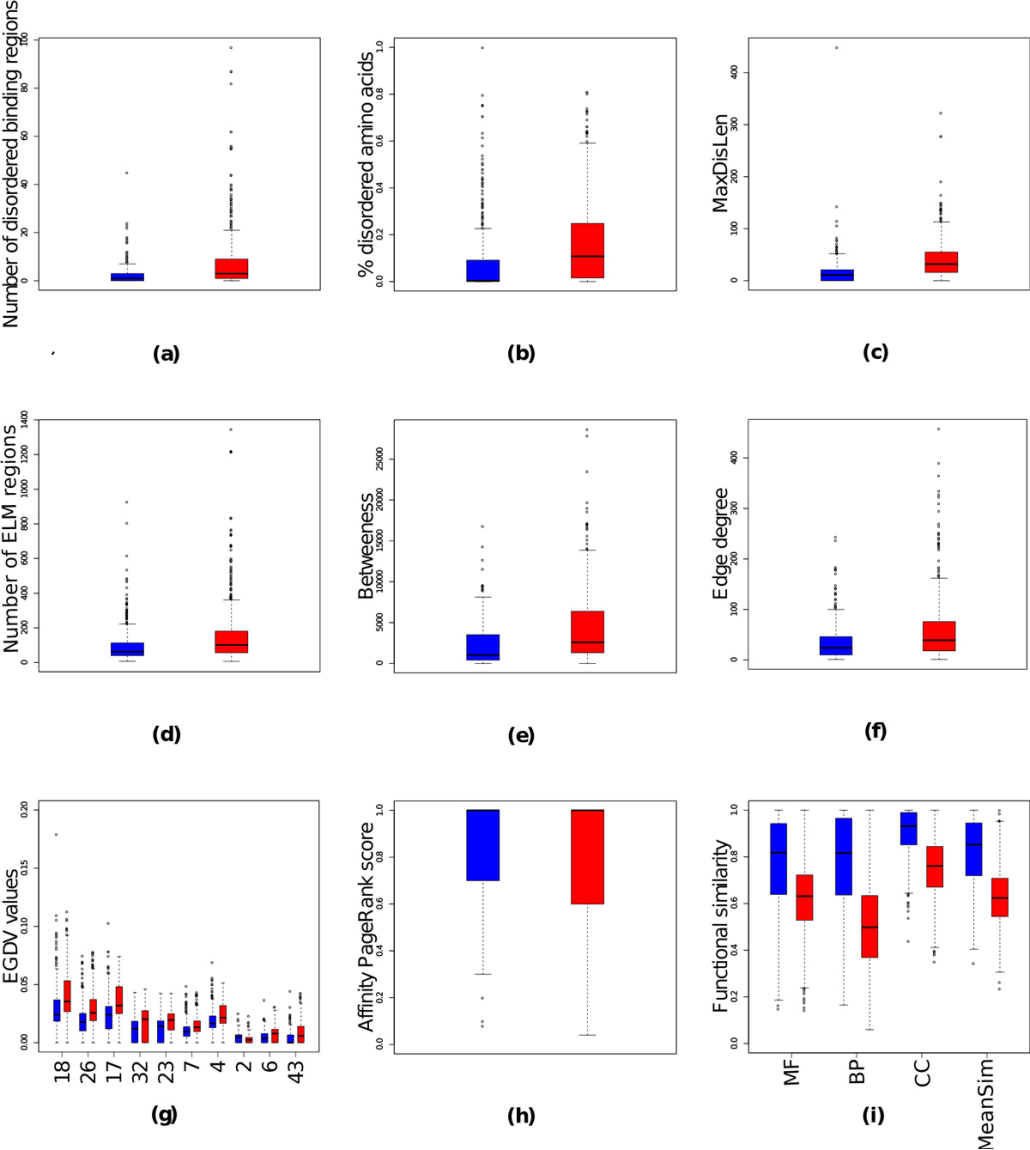
**Boxplot distributions of features in obligate (red) and non-obligate (blue) interactions**. For the number of disordered binding regions, the fraction of disordered amino acids, and the number of found ELM both values for protein A and B are combined into one distribution. For EGDV (g) only top 10 features with the lowest P value are plotted.

**Figure 4:**
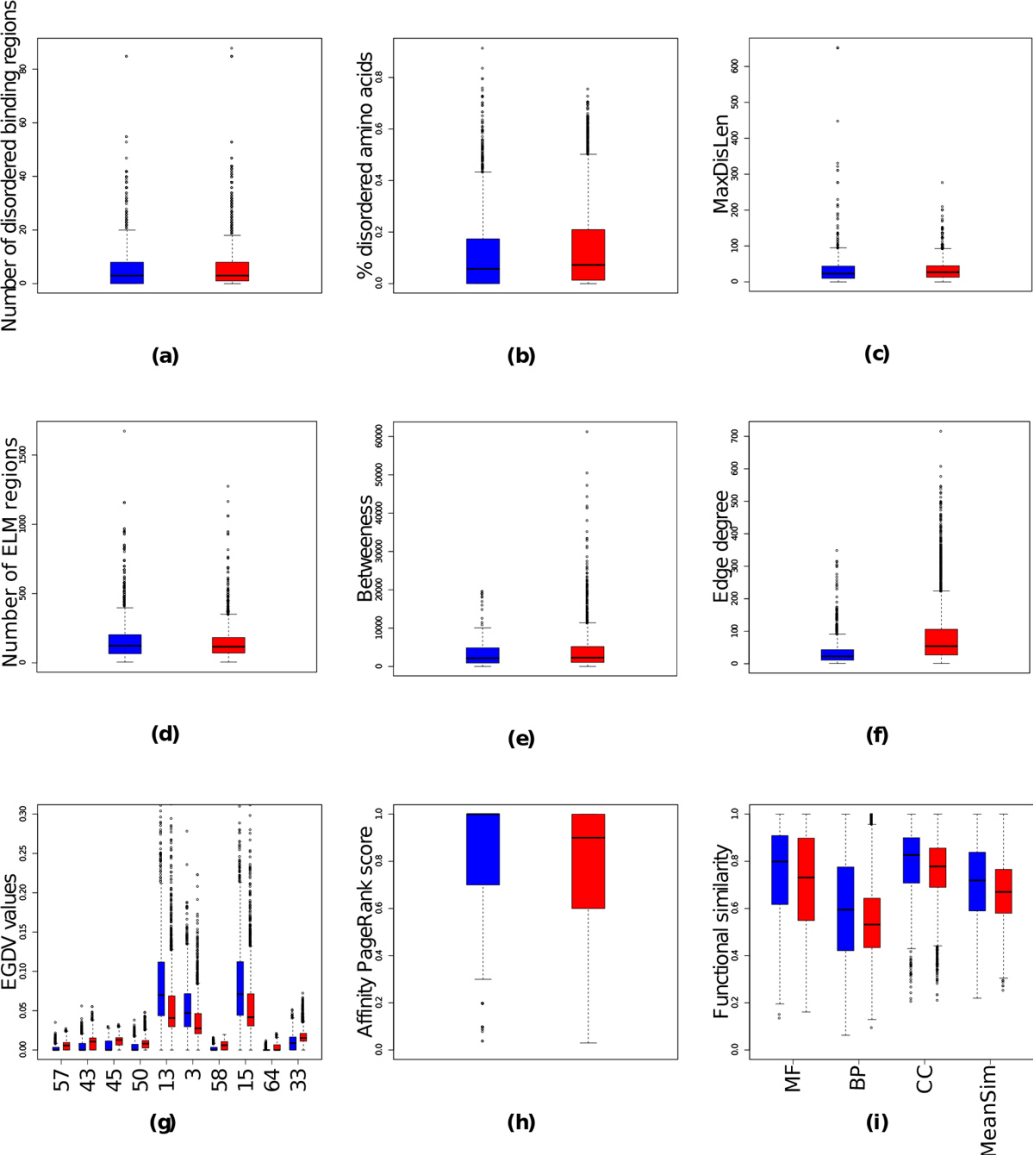
**Boxplot distributions of features in simultaneously possible (red) and mutually exclusive (blue) interactions**. For the number of disordered binding regions, the fraction of disordered amino acids, and the number of found ELM both values for protein A and B are combined into one distribution. For EGDV (g) only top 10 features with the lowest P value are plotted.

We started by searching for features (see Table 4 for abbreviations used) that are best discriminant for each of the two classification problems addressed in this work - obligate *vs *non-obligate interactions and SP *vs *ME interactions (Figures 3, 4) - and ranking features based on their Wilcoxon ranked-sum test P-value (for the top 40 features see Tables 5, 6, and for the bottom 40 features see Additional file 1). For a better overview we grouped features into three distinct sets - functional similarity (BP, CC, MF, MeanSim; total of 4 features), sequence features (ELM, disorderedness, total of 8 features), and network features (degree, Affinity PageRank, betweenes, EGDV, total of 71 features).

**Table 4:** Features used for machine learning

Name	Abbreviation
Sequence based features	

Number of found short linear eukaryotic motifs in protein A	elmA

Number of found short linear eukaryotic motifs in protein B	elmB

Number of disordered binding regions in protein A	DisRegionsA

Number of disordered binding regions in protein B	DisRegionsB

Fraction of disordered Amino Acids in protein A	FracDisASA

Fraction of disordered Amino Acids in protein B	FracDisASB

Length of the longest disordered binding regions in both proteins	MaxDisLen

Network based features	

Degree	Degree

Betweeness of the interactions	Betweeness

Affinity page rank score for the interactions	APR

EGDV values for orbit *n *= {1,2,3, ..., 69}	1, 2, 3, ..., 69

Functional similarity based features	

Functional similarity based on cellular component GO terms	CC

Functional similarity based on biological process GO terms	BP

Functional similarity based on molecular function GO terms	MF

Mean of CC, BP, and MF values.	MeanSim

**Table 5:** Ranking of the top 40 features for the obligate and non-obligate classes based on their Wilcoxon ranked sum test P-values.

Rank	Feature name	Mean obligate	Mean non-obligate	P-value	Rank	Feature name	Mean obligate	Mean non-obligate	P-value
1	MeanSim	0.816	0.63	7.2e-48	21	33	0.0095	0.0148	2e-08

2	CC	0.9	0.748	1.7e-47	22	45	0.0049	0.00841	6.5e-08

3	BP	0.768	0.517	2.7e-41	23	50	0.0040	0.00737	8.6e-08

4	DisRegions	2.17	7.27	9.3e-39	24	57	0.0016	0.00395	2.7e-07

5	FracDisAS	0.0737	0.163	2.4e-35	25	30	0.0224	0.0281	5.3e-07

6	MaxDisLen	17.5	42.2	1.9e-31	26	Degree	35.4	59	7.7e-07

7	MF	0.779	0.626	4.3e-24	27	31	0.015	0.0216	2.1e-06

8	ELM	96.2	151	1.3e-18	28	39	0.0155	0.0193	3.9e-05

9	Betweeness	2463	4315	4.8e-16	29	12	0.0027	0.00132	0.0001

10	18	0.0293	0.04	6.5e-14	30	25	0.0225	0.0148	0.0001

11	26	0.019	0.0274	7.8e-13	31	8	0.0122	0.0082	0.0001

12	17	0.0239	0.0326	7.4e-12	32	52	0.0079	0.00432	0.0001

13	32	0.0109	0.0177	4.2e-11	33	68	0.0017	0.00065	0.0001

14	23	0.0122	0.0181	4.3e-11	34	51	0.0065	0.00398	0.0007

15	7	0.0105	0.0141	2.1e-09	35	41	0.0082	0.00484	0.0008

16	4	0.0173	0.0222	6.3e-09	36	20	0.0298	0.0353	0.001

17	2	0.0043	0.00258	6.9e-09	37	24	0.0216	0.0159	0.0017

18	6	0.0045	0.00752	8e-09	38	44	0.0058	0.00858	0.0022

19	43	0.0037	0.00804	1e-08	39	11	0.0031	0.00161	0.0026

20	5	0.0267	0.0323	1.2e-08	40	49	0.0027	0.00114	0.0028

The numbers in the name column refer to EGDV values for orbits (see Table 4). For the number of disordered binding regions, fraction of disordered amino acids, and ELM both values for protein A and B are combined into one distribution which has two values for each interaction. The Wilcoxon P-value is then calculated for this distribution.

**Table 6:** Ranking of the top 40 features for the simultaneously possible and mutually exclusive classes based on their Wilcoxon ranked sum test P-values.

Rank	Feature name	Mean SP	Mean ME	P-value	Rank	Feature name	Mean SP	Mean ME	P-value
1	Degree	36.6	86.9	2.8e-106	21	42	0.00631	0.0108	8.2e-45

2	57	0.00207	0.00584	2.5e-95	22	16	0.0657	0.0502	1.2e-44

3	43	0.00486	0.0104	9.8e-86	23	47	0.00333	0.00622	2.7e-44

4	45	0.006	0.0113	5.2e-82	24	10	0.00372	0.00619	1.5e-43

5	50	0.00407	0.00853	3.6e-81	25	36	0.00772	0.0125	1.3e-41

6	13	0.0895	0.0552	9.5e-79	26	65	0.00099	0.00263	7.6e-38

7	3	0.0556	0.0363	1.2e-76	27	32	0.0145	0.0206	1.4e-31

8	58	0.00211	0.00563	1.4e-76	28	66	0.00116	0.00263	5.9e-29

9	15	0.0871	0.0558	3.8e-76	29	34	0.00912	0.0136	1.7e-26

10	64	0.00076	0.00315	3.9e-74	30	38	0.00576	0.00866	3.4e-26

11	33	0.00999	0.0166	2.1e-66	31	55	0.00267	0.00481	2.3e-23

12	48	0.00309	0.00716	3.7e-66	32	23	0.0152	0.0197	1.1e-22

13	54	0.0033	0.00707	3.7e-65	33	61	0.00211	0.00397	6e-22

14	22	0.0491	0.0344	2.8e-63	34	40	0.00921	0.0133	9e-21

15	60	0.00176	0.00444	9.9e-61	35	59	0.00186	0.003	9.8e-21

16	56	0.00187	0.0048	7.1e-58	36	7	0.0117	0.0144	5.6e-20

17	6	0.00608	0.00917	9e-53	37	62	0.00172	0.0029	2.1e-19

18	44	0.00651	0.0107	2.4e-50	38	67	0.00076	0.00156	1.1e-17

19	1	0.0265	0.0199	3.4e-48	39	14	0.0474	0.039	2.4e-17

20	53	0.0044	0.00793	2.3e-46	40	46	0.00712	0.00968	2.2e-16

The numbers in the name column refer to EGDV values for orbits (see Table 4). For the number of disordered binding regions, fraction of disordered amino acids, and ELM both values for protein A and B are combined into one distribution which has two values for each interaction. The Wilcoxon P-value is then calculated for this distribution.

#### Sequence based features

In this work we evaluated two sequence features - number of ELMs and number of predicted disordered regions. On average non-obligate interactions tend to have almost three times as many disordered regions than obligate interactions (rank 4 in Table 5, Figure 3a) and the proteins that participate in non-obligate interactions have a considerably higher fraction of disordered amino acids (rank 5 in Table 5, Figure 3b). Furthermore, the longest binding regions associated with non-obligate interactions tend to be twice as long as those in obligate interactions (rank 6 in Table 5, Figure 3c). These results are in line with recent reports, which state that proteins involved into non-obligate interactions tend to be more disordered than those associated with obligate interactions [59]. We also found that non-obligate interactions tend to have more ELM regions (rank 8 in Table 5, Figure 3d), which agrees with the notion that ELM primarily mediate weak transient interactions occurring in signaling [60].

Proteins involved in SP interactions tend to be more disordered than those in mutually exclusive interactions (Table 6, Figure 4a-c), presumably because simultaneously possible interactors undergo stronger conformational changes upon binding their partners than mutually exclusive interactors [61]. At the same time we do not find any significant difference in the distribution of ELMs in SP and ME interactions (P-value 1, rank 73 in Table S2, Figure 4d).

#### Network based features

Network based features do not play a significant role in distinguishing between obligate and non-obligate interactions. Overall, they performed poorly (Figure 3e-f), with only betweeness showing a high rank in Table 5. However we do find that orbits 2, 25, 8, 52, and 68 (rank 17, 30, 31, 32, and 33 in Table 6) located inside cliques (Figure 1, section 2.5.1) are enriched in obligate interactions while orbits 18, 26, 17, 32, and 23 describing hub-like proteins (Figure 1, section 2.5.1) are enriched in non-obligate interactions (rank in 10-14 Table 6, Figure 3g). In particular, the orbit number 68, the five clique, occurs three times more often in obligate interactions than in non-obligate interactions (P-value 0.00001, rank 33 in Table 6), yet the signal is too weak to distinguish those classes efficiently. This observation is compatible with the fact that obligate interactions are permanent and usually occur in functional modules corresponding to tightly connected clusters in interaction networks [62]. Indeed, we observed slightly larger APR values for obligate interactions (Figure 3h) that for non-obligate interactions.

In contrast, network topology differs greatly between SP and ME interactions, since there is a physical limit to how many interaction partners can simultaneously bind to a protein [22]. While we observed no significant difference for betweeness (P-value 1, rank 70 in Table S2, Figure 4e), degree is the best feature to separate these two classes (rank 1 in Table 6, Figure 4f). There are also differences in local topology, with the orbits 13, 3, 15, 22, and 6 enriched in SP interactions and orbits 57, 43, 45, 50, 58 being more prominent in ME interactions (Figure 4g). ME interactions were enriched in orbits describing bottlenecks, with 58 being the only exception (Figure 1, section 2.5.1), which implies that ME interactions are key connectors in the interaction network and that at least one of the two interacting proteins has a higher chance to be an essential gene [40]. In contrast, SP interactions prefer sparsely connected orbits (Figure 1, section 2.5.1), probably due to the physical limits of binding multiple partners simultaneously. Similar to obligate interactions, SP interactions tend to have larger APR values (Figure 3h).

#### Functional similarity

For the obligate/non-obligate classification the most significant P-values were reached with functional similarity features (ranks 1-3 in Table 5, Figure 3i). This is caused by the fact that all obligate interactions are permanent while the majority of non-obligate interactions are transient [4]. The only permanent non-obligate interactions are antibody-antigen and enzyme-inhibitor interactions. Further work is needed to distinguish those interactions from signaling and receptor-ligand interactions, which would open up the possibility of classifying interactions as permanent or transient and also distinguishing between strong and weak interactions.

For the SP/ME classification we only find a weak correlation with functional similarity (ranks 48, 53, 60 62 in Table 6, Figure 4i). However, all functional similarity features had a P-value of less than 0.05 (significant level). Thus, we expect it to play at least some part in the classification.

#### Predictor evaluation

As an additional evaluation method we trained a random forest classifier (RUSBoost ratio 0.37 for obligate/non-obligate, and 0.31 for SP/ME; see Additional file 3 for justification of these values) with either all features or only with features from each individual group (functional similarity, network based features, sequence based features). In a 10-fold cross-validation the auROC values for obligate/non-obligate classification were 0.881, 0.810, 0.822, and 0.772 for all features, for functional similarity, sequence features, and network features, respectively.

Analogously, we performed the same analysis for simultaneously possible and mutually exclusive interactions. The random forest auROC values (RUSBoost ratio 0.31) were 0.851, 0.657, 0.806, and 0.808 for all features, for functional similarity, sequence features, and network features, respectively. Using either disordered features or ELM features separately we achieved an auROC of 0.75 and 0.66, respectively. However, when both disordered and ELM features were utilized the auROC was substantially higher - 0.806 - underlying the importance of cross-talk between these two groups of biological properties.

### Predictor evaluation

We preformed an extensive analysis and benchmarking of both the obligate/non-obligate and SP/ME classifiers, which can be found in the Additional file 3. Overall we achieved an auROC of 0.881 and 0.851 for obligate/non-obligate and SP/ME classification, respectively. The F-measure values were 0.56, 0.88, 0.71, and 0.85 for SP, ME, obligate, and non-obligate classification, respectively. Notably, these results solely based on sequence and network information are only marginally worse than 3D-structure derived predictions by NOXClass [17].

### Large scale classification of protein interactions

We applied our method to classify 13978 HeLa and 83788 iRefIndex protein interactions as either obligate or non-obligate as well as either SP or ME. Each interaction was also attributed to one of the four class combinations - obligate and SP, obligate and ME, non-obligate and SP, or non-obligate and ME - and assigned two confidence values - one for the SP/ME classification and one for the obligate/non-obligate classification.

**Figure 5:**
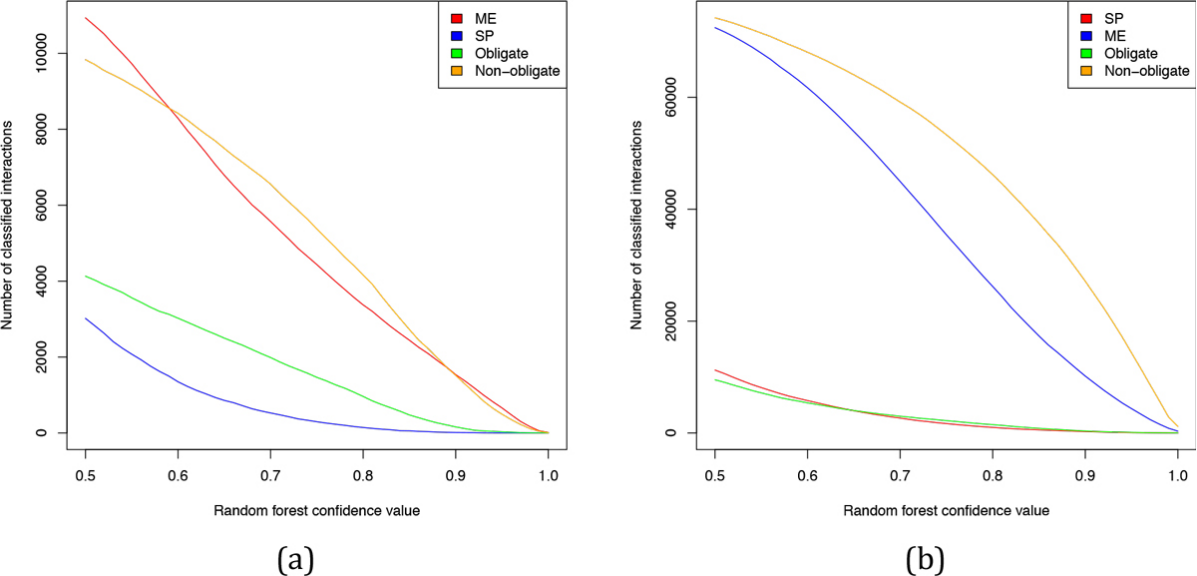
**Number of classified interactions for each class for various random forest confidence cutofs in the HeLa dataset (a) and iRefIndex (b) dataset**.

**Figure 6:**
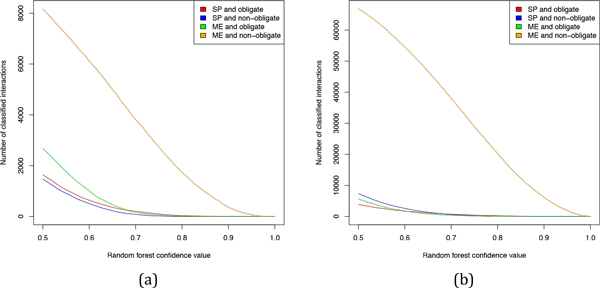
**Number of classified interactions for all possible class combinations (SP and obligate, SP and non-obligate, ME and obligate, and ME and non-obligate) in the HeLa (a) and iRefIndex (b) dataset**.

We analyzed the number of classified interactions for each class and class combination for various random forest confidence values (Figure 5, 6). Note that we ignored cases where the classifier was indecisive (i.e. confidence 0.5 for both classes). The number of classified cases declines with increasing stringency of the classifier. For example, at the random forest confidence value of 0.7 two thirds of interactions get classified and around 10% are left at the 0.9 threshold.

**Figure 7:**
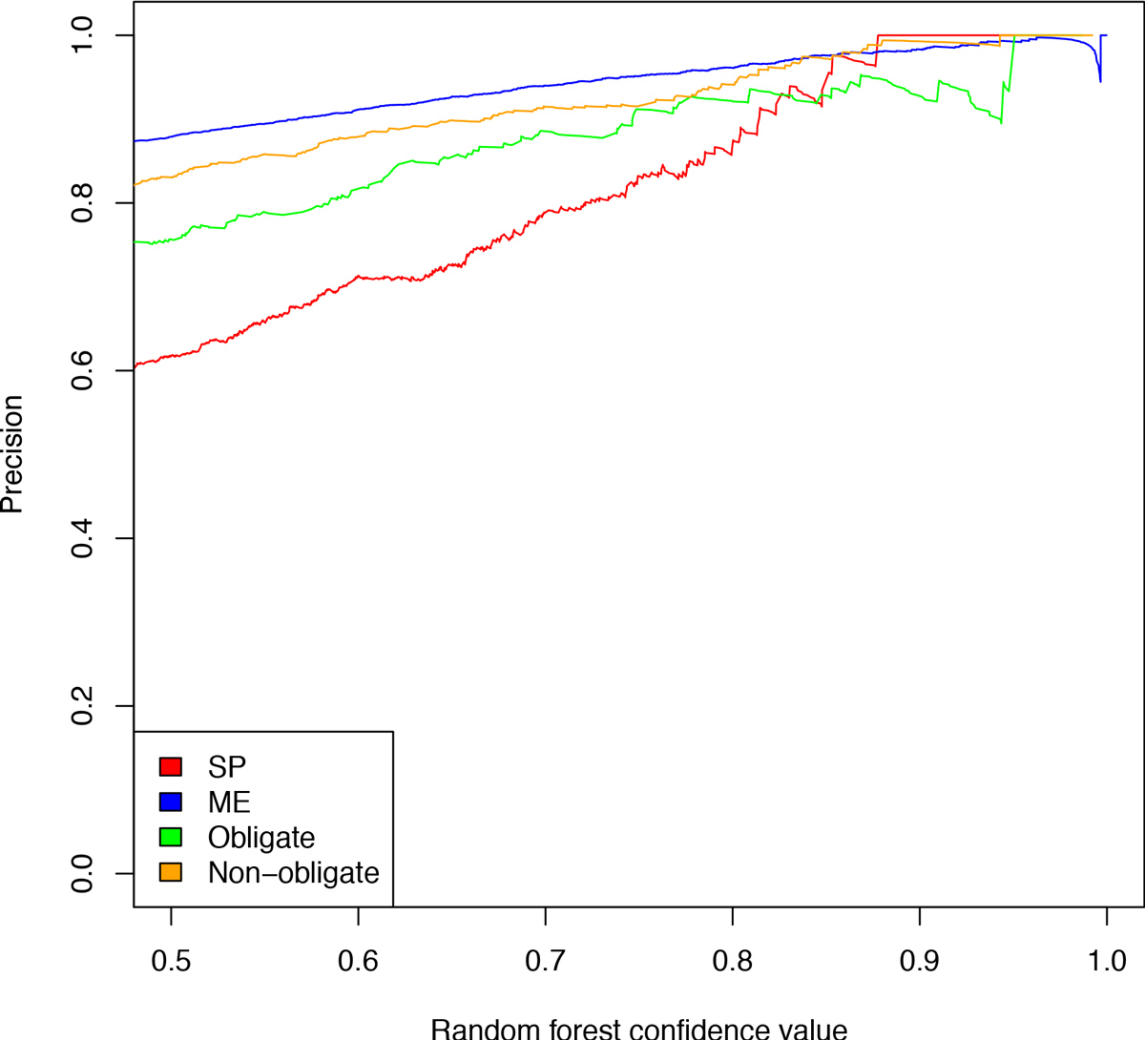
**Dependence of the classifier precision on random forest confidence value cutoff in a 10-fold cross-validation**.

What is the optimal threshold for the random forest confidence values? As seen in Figure 7 the classifier precision, determined by 10-fold cross-validation, is positively correlated with the random forest confidence value cut-off. In particular, at the cutoff value of 0.6 the classifier precision is 0.72, 0.9, 0.83, and 0.88 for SP, ME, obligate, and non-obligate classification, respectively. In other words, it achieves precision of over 0.8 for each classification problem, except for SP classification. However, since 21% of the SP/ME interactions are SP, a random SP classifier achieves a precision of 0.21, which means that our classifier is considerably better than a random classifier. Furthermore, the confidence value cutoff of 0.6 seems an acceptable trade off between precision and the number of classified interactions. Another reason to choose 0.6 as a cut-off value is that it guarantees the difference in confidence values between the opposing classes of at least 0.2. Note that confidence values are calculated by weighted majority voting. This means that at least 60% of the weighted random forest trees decided in favor of the chosen class and at most 40% for the opposing class, which implies that the classifier decision is based on a distinct majority.

For random forest confidence values >= 0.6 the total of 638 and 506 HeLa interactions as well as 1747 and 2620 iRefIndex interactions were classified as SP/obligate and SP/non-obligate, respectively. The total of 1010 and 6118 HeLa interactions were classified as ME/obligate and ME/non-obligate, respectively, while for the iRefIndex dataset the corresponding numbers were 1772 and 54580.

It was recently suggested that SP interactions are mostly permanent and ME interactions are mostly transient [22]. As discussed above transient interactions are by definition non-obligate while permanent interactions are mostly obligate. In line with the results reported in [22] most of the ME interactions were classified as non-obligate both in the HeLa and iRefIndex datasets, presumably because proteins involved in ME interactions compete for the same binding side, which is only possible when the interactions are non-obligate. However, we found that 44% of the SP interactions in the HeLa dataset and 59% of the SP interactions in the iRefIndex were classified as non-obligate (compare Figures 5 and 6). This result implies that a multimeric protein complex can either exist as a stable compound throughout its entire lifetime or it can dynamically form and dissolve during its lifetime. An example for a non-obligate multimeric protein complex are coat proteins involved in formation of molecular vesicles. The coat proteins associate together to form the coat of the molecular vesicle and upon delivering their payload they dissolve again from each other.

**Figure 8:**
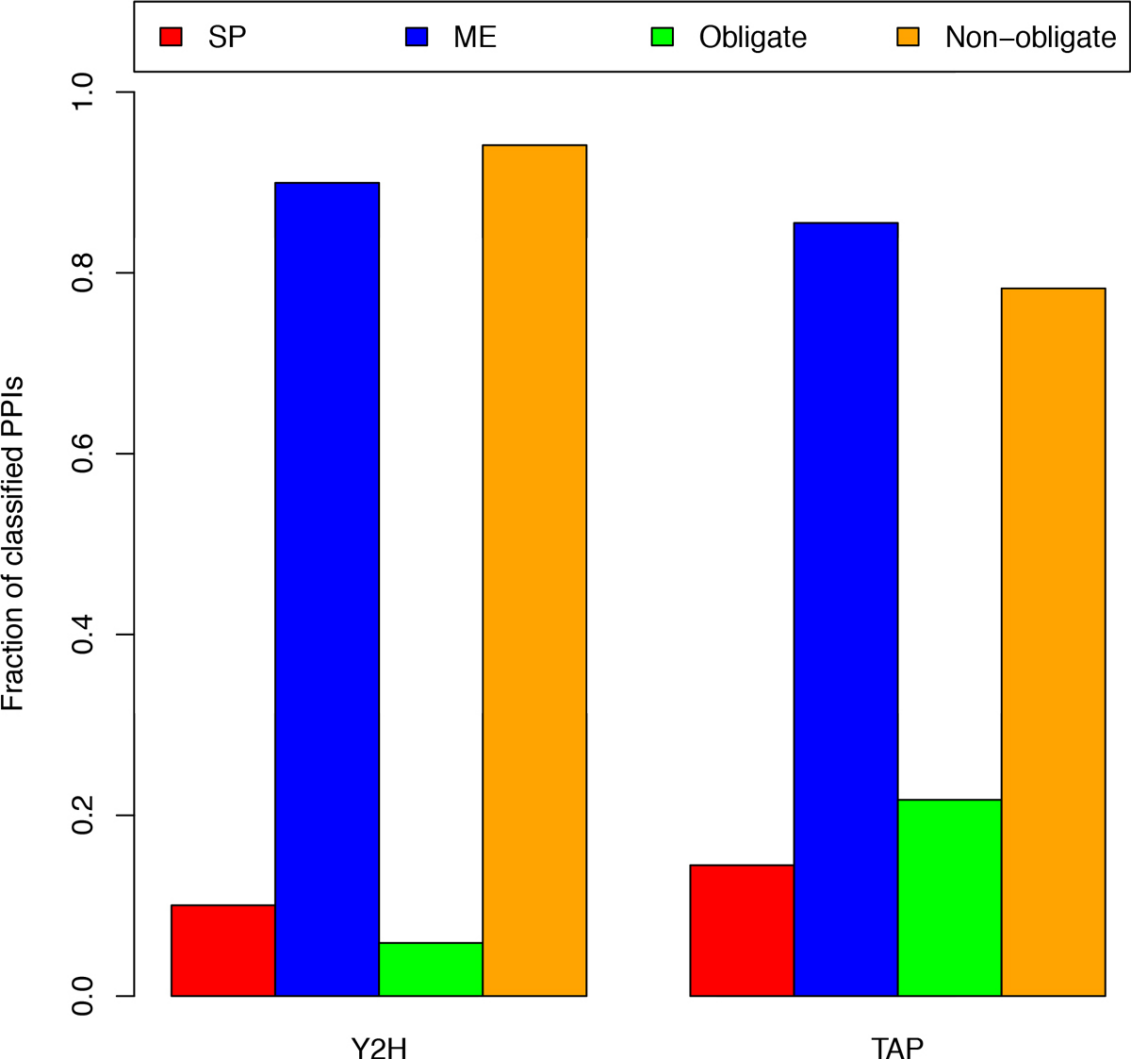
**Class distributions for predicted protein interactions measured by the yeast two hybrid (Y2H) and tandem affinity purification (TAP) methods**.

We also evaluated the classification results for iRefIndex interactions measured by different experimental methods, focusing on yeast two hybrid (Y2H) essay and tandem affinity purification (TAP). It was suggested that TAP has a preference for detecting obligate interactions while Y2H has no bias towards obligate or non-obligate interactions [3]. Indeed, as shown in Figure 8, interactions determined by TAP get classified as obligate three times more often that those measured by Y2H. At the same time the fraction of SP interactions increases by 40% from 0.10 in Y2H to 0.14 in TAP, in line with the previous observation that around half of the SP interactions are also obligate.

#### Protein complex analysis

We further applied our method to classify all intra- and inter-complex interactions in the CORUM and HeLa datasets (see section Protein complex data). The overlap between different class combinations in terms of GO categories associated with them is very low, implying that each interaction type is intrinsic for a distinct set of cellular functions (see Additional file 4). As expected, most of the inter-complex interactions in each dataset (CORUM, HeLa) were classified as ME/non-obligate, indicating that protein complexes interact mostly transiently with each other.

**Figure 9:**
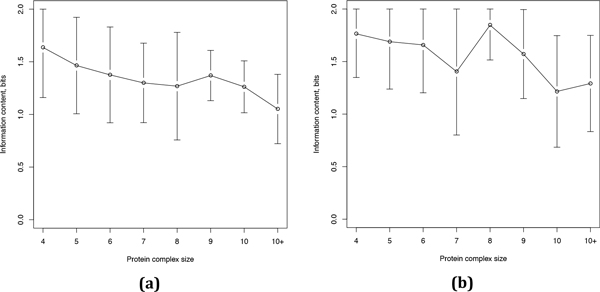
**Information content *vs *protein complex size in the CORUM (a) and HeLa (b) datasets**. Dots indicate the mean value of the information content and the error bars show its standard deviation.

**Figure 10:**
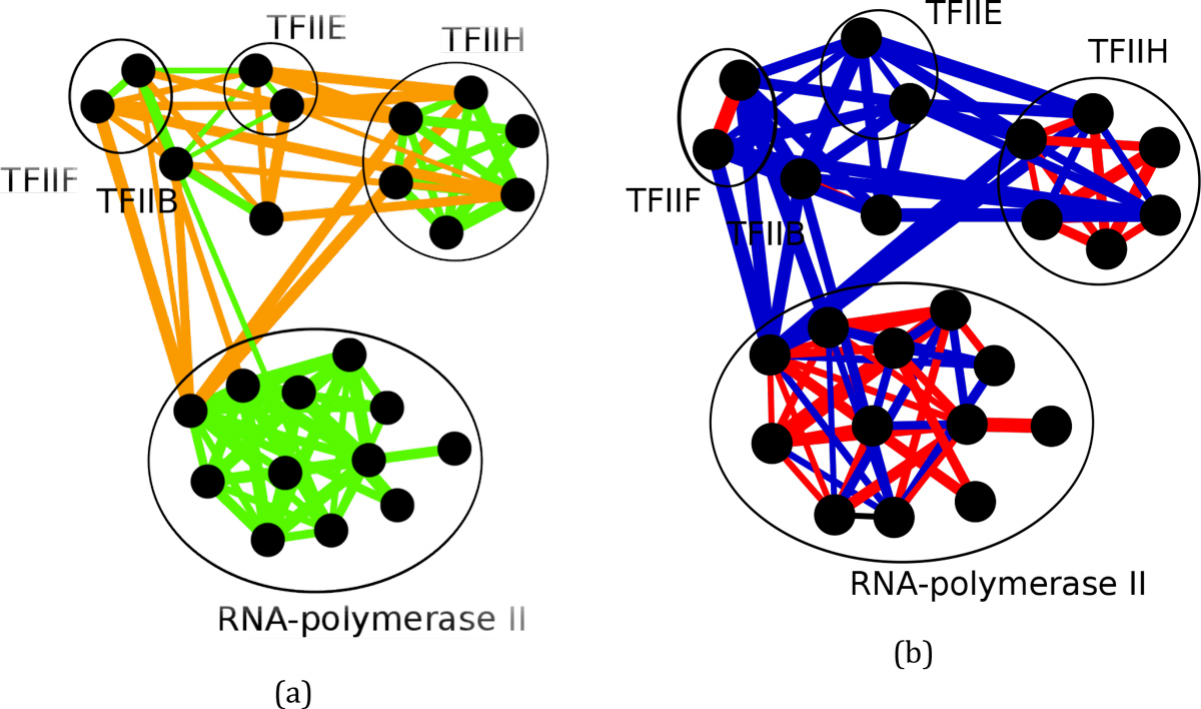
**Protein interactions within the RNA polymerase II holoenzyme complex (CORUM ID: 103) classified as obligate (green) *vs *non-obligate (orange) (a) and SP (red) *vs *ME (blue) (b)**.

With regard to intra-complex interactions we observed that small protein complexes possess high information content and thus tend to be enriched in just one interaction type (Figure 9). Larger protein complexes generally display increased diversity in terms of interaction types (except for complexes of size 8 in the HeLa dataset for which the sample size is very small), probably because they may contain functionally specialized subcomplexes, each with its own prevailing interaction type. For example, RNA polymerase II and the transcription factor TFIIH form an obligate/SP sub-compartment while TFEII, TFFII, and TFIIB are mostly involved in non-obligate/ME interactions, and the interactions between TFHII and the RNA polymerase II are also mostly non-obligate/ME (Figure 10).

**Figure 11:**
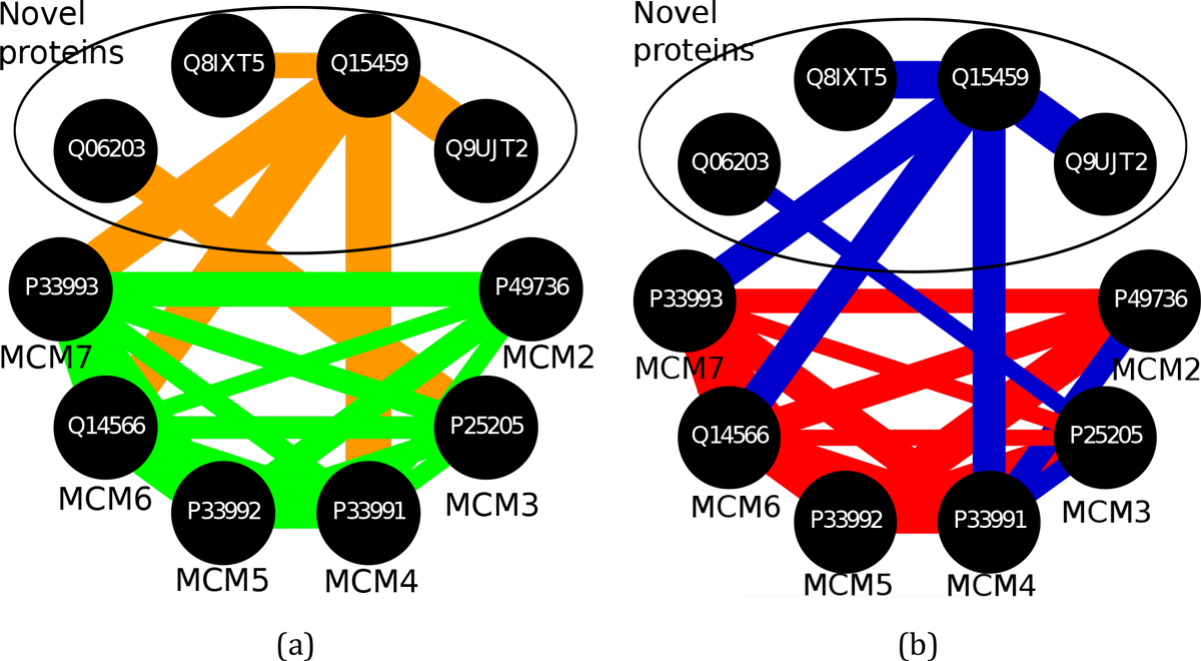
**Protein interactions within the mini-chromosome maintenance (MCM) complex (HeLa ID: 587) classified as obligate (green) *vs *non-obligate (orange) and SP (red) *vs *ME (blue) (b)**. Uniprot accession numbers are shown for each protein. Additionally gene names are shown for members of the CORUM MCM complex.

Knowledge about interaction types can be instrumental for assessing the quality of protein complexes derived by computational methods. For example, the predicted mini-chromosome maintenance (MCM) complex (HeLa ID 587, Figure 11) consists of an obligate/SP part and a non-obligate/ME part. The obligate part exactly matches the CORUM MCM complex (CORUM ID 387), which is essential for DNA replication, initiation, and elongation in eukaryotic cells. The non-obligate/ME part is a novel addition to the MCM complex, which consist of the following proteins: amidophosphoribosyltransferase, RNA-binding protein 12B, splicing factor 3A subunit, and testis-specific serine kinase substrate. These proteins do not have any biological function associated with DNA replication, initiation, and elongation and it is probably safe to assume that they constitute false positive predictions added in the predicted HeLa complex to the manually verified CORUM complex.

**Figure 12:**
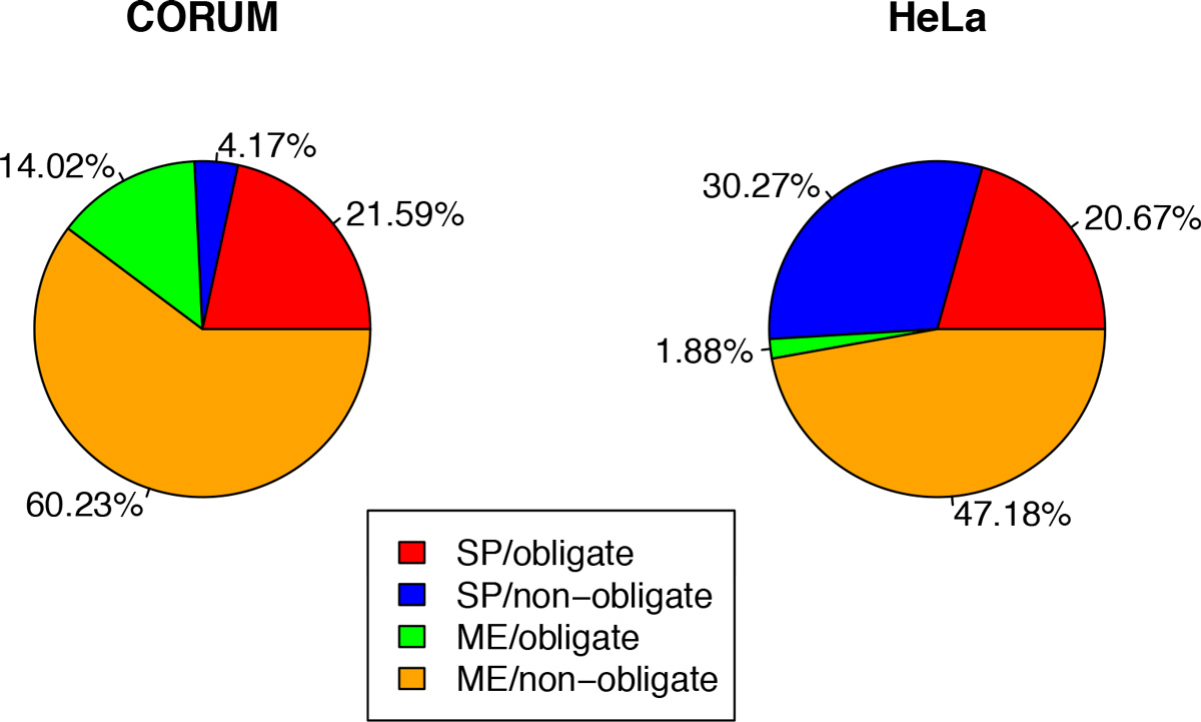
**Fraction of enriched protein complexes in each dataset**.

We defined a protein cluster to be enriched in a given interaction type when it constituted at least 50% of the intra-complex interactions and plotted the fraction protein complexes enriched in each interaction type (Figure 12). Both in the HeLa and in the CORUM datasets most of the protein complexes are enriched in ME/non-obligate interactions due to the fact that most of the ME interactions are non-obligate and the latter are frequently involved in intracellular signal transduction [2]. Correspondingly, ME/non-obligate interactions are enriched in GO terms associated with biological process regulation (see Additional file 4). Furthermore, around 50% of the protein complexes in the HeLa dataset are enriched in SP interactions whereas in the CORUM dataset only 25% of the complexes are SP-heavy. We speculate that the reason for this discrepancy lies in the somewhat different nature of these two datasets. The CORUM dataset used in this work was generated by overlaying multi-protein complexes described in the CORUM database with the binary interactions from the iRefIndex resource, while the HeLa dataset was derived by its authors by applying the CLusterOne method to a high confidence PPI network.

## Conclusions

We report PiType, a novel technique for classifying protein interactions into obligate/non-obligate as well as into SP/ME based exclusively on sequence and network information. In contrast to previous work that relied on known 3D structures of proteins our method is suitable for large-scale characterization of interaction data. In particular it can be applied to improve protein complex prediction. Its performance is comparable with that of the structure-based classifiers, achieving an auROC of at least 80% and a F-measure close to 80% in a nested cross-fold validation. PiType is available at http://webclu.bio.wzw.tum.de/PiType/PiType.zip.

We have conducted a thorough investigation of sequence and network features characteristic for each type of interactions. Proteins involved in non-obligate interactions tend to have more disordered regions and short linear eukaryotic motifs than obligate interactors. Non-obligate interaction partners are also less functionally similar than obligate interaction partners. These results are in line with previous observations [3] (note that the majority of non-obligate interactions are transient). Likewise, SP interactors are more disordered than ME interactors, most likely because the former undergo stronger conformational changes upon binding their partners [61]. By analyzing edge graphlet degree vectors (EGDV) we identified network contexts in which interactions of different types typically occur. Obligate interactions are enriched in orbits associated with tightly connected clusters, whereas non-obligate interactions are frequently found in orbits characteristic for network hubs. EGDV analysis also revealed that ME interactions are enriched in orbits describing bottlenecks, key network elements with high betweenness. In contrast, we observed that orbits with a low edge degree (i.e. 13, 3, 15, 22, and 6; see Figure 1) are more prominent in SP interactions for still unclear reasons.

## Abbreviations

APR: PageRank affinity; auROC: area under receiver operating curve; EGDV: edge graphlet degree vector; ELM: short linear eukaryotic motifs; GO: gene ontology; KNN: k nearest neighbors; MCM: mini-chromosome maintenance; ME: mutually exclusive; PR: precision-recall; ROC: receiver operating characteristic; SIN: structural interaction network; SP: simultaneously possible; SVM: support vector machine; TAP: tandem affinity purification; Y2H: yeast two hybrid.

## Authors' contributions

F.G. and D.F. jointly conceived the study. F.G. carried out the analysis of the data. D.F. supervised the work. F.G. and D.F. wrote and revised the manuscript.

## Competing interests

The authors declare that they have no competing interests.

## Endnote

^a ^Definition for raw interactions and non-redundant interactions are taken from http://wiki.thebiogrid.org/doku.php/statistics.

## Supplementary Material

Additional file 1 **Bottom 40 ranked features for both SP/ME classification and obligate/non-obligate classifications**. Click here for file

Additional file 2 **Benchmarking methods for both SP/ME classification and obligate/non-obligate classifications**. Click here for file

Additional file 3 **Benchmarking results for both SP/ME classification and obligate/non-obligate classifications**. Click here for file

Additional file 4 **GO enrichment results**. Click here for file

## Declarations

The *BMC Systems Biology *publication fee was paid by the DFG International Research Training Group 'Regulation and Evolution of Cellular Systems' (GRK 1563).

This article has been published as part of *BMC Systems Biology *Volume 7 Supplement 6, 2013: Selected articles from the 24th International Conference on Genome Informatics (GIW2013). The full contents of the supplement are available online at http://www.biomedcentral.com/bmcsystbiol/supplements/7/S6.
