# Supplementary material for: Prediction of protein interaction types based on sequence and network features
Source: BMC Syst Biol. 2013 Dec 13;7(Suppl 6):S5. doi: 10.1186/1752-0509-7-S6-S5 (PMC4029746; doi:10.1186/1752-0509-7-S6-S5)
Supplement: Additional file 1 — Bottom 40 ranked features for both SP/ME classification and obligate/non-obligate classifications. [file 1752-0509-7-S6-S5-S1.PDF]

Additional file 1

RUSBoost

We chose the RUSBoost algorithm [1] for alleviating class imbalance as it reportedly outperforms many other approaches [2]. The basic idea of RUSBoost is to combine boosting with random under-sampling. Boosting combines several weak learners into one strong learner. The weak learners are those trained on a subset of the training set, which is generated by random sampling with replacement. During the sampling process only two thirds of the data are used to train the weak learner while the remaining third can be used to evaluate and appropriately weight the classifier. These steps are repeated until a number of weak learners are created whose weighted combination constitutes the strong learner. The RUSBoost algorithm applies a modified resampling step. Instead of randomly subsampling the complete dataset it removes members from the majority class until a given ratio between the minority and the majority class is reached. The disadvantage of this approach is that the weak learners are always trained on the same members of the minority class. We therefore altered the RUSBoost algorithm in such a way that the members of the majority class are first removed, followed by one round of boosting on the remaining data. This process of resampling and boosting is repeated multiple times.

Feature selection

The random forest classifier we used in this work (see below) applies internal estimates to measure variable importance. Such classifier is therefore intrinsically robust even for very noisy data, so that no appreciable improvement in evaluation metrics through feature selection would be expected [3]. The implicit feature selection process is hardly traceable for the user, especially because random forest is an ensemble of multiple decision trees and one feature can be part of multiple decision rules.

We therefore conducted a separate investigation of feature importance for each classification problem using three different methods.

- Information gain. Features were ranked based on their information gain and the twenty best features were selected.
- Genetic algorithm. Feature subsets were searched and created with the genetic algorithm. Each feature subset was evaluated according to the performance of the random forest classifier on the subset.
- Correlation-based feature selection. Here the results of two rounds of greedy feature selection with correlation based evaluation - a) forward selection (start with no features and add features until convergence, i.e. when no improvement is achieved after five iterations), and b) backward selection (start with all features and remove features until convergence, as above) - are combined, retaining only those features selected in both rounds.

Naïve classifier

In order to have a better understanding of how well our method performs we would ideally need to compare it to other available predictors. However, since no other sequence or network-based predictors currently exist we compare our classifier to a naïve version of itself. This naïve version is a baseline RUSBoosted random forest classifier trained with only one feature. This feature is the number of proteins in an interacting pair annotated with the GO term "protein complex" or any children thereof and can thus take one of the three values 0, 1, or 2. We expect this feature to distinguish between those interacting protein pairs that are part of a protein complex and are thus presumably involved in an obligate interaction as opposed to those interactors that are not known to be part of a complex.

Performance measures

We applied nested–fold cross–validation [4] to evaluate the degree of overfitting of our predictor. Data were divided into three equal sized disjoint sets and all possible permutations of these datasets ({1,2,3}, {1,3,2}, etc) were generated. The first set was then used to train the feature selection process, the second set to train the predictor, and the third set to evaluate the predictor. In addition to the nested-fold cross-validation we also evaluated the predictor's overall performance by a regular 10–fold cross–validation procedure. We further validated our method on a holdout set of obligate and non-obligate interactions, which involved training the classifier on the NOXclass predicted data and evaluation on the combined Zhu and Mintseris dataset.

The following standard cross–validation performance measures were employed:

- Precision: the fraction of predicted interactions that are correctly classified:

$$Precision = \frac{TP}{TP+FP}$$

- Recall or True positive rate: the fraction of interactions in the evaluation set that are correctly classified:

$$Recall = True\ positive\ rate = \frac{TP}{TP + FN}$$

- False positive rate:

$$False\ positive\ rate = \frac{FP}{FP + TN}$$

- F–measure: harmonic mean of precision and recall

$$F - measure = \frac{2\ Precision * Recall}{Precision + Recall}$$

We also plotted the receiver operating characteristic (ROC) [5] and estimated the overall performance of the classifier based on the area under the ROC curve (auROC) [6] with the help of the R package [7]. In addition we utilized Precision–Recall (PR) curves to assess the precision of our classifier for various recall values [8]. These Precision–Recall values are generated by applying various thresholds to the predictor's confidence value. Furthermore we used the R implementation of the Wilcoxon ranked sum test [9], which assesses whether one of two samples tends to have larger sample values than the other one. This test can be used to rank features with different distributions, since it is independent of the feature distribution.

Cross species evaluation

In order to assess the generalization power of our method across different taxonomic kingdoms we split protein interaction data (see section 2.4) into three separate datasets for human, yeast, and *E. coli*. We then conducted a 10–fold cross–validation of three classifiers trained on individual organism-specific datasets and compared the resulting auROC values to those obtained in all possible cross-species validation experiments in which a classifier trained on data from one organism is evaluated on data for another organism.

1. Seiffert, C., et al., *RUSBoost: A hybrid approach to alleviating class imbalance.* Systems, Man and Cybernetics, Part A: Systems and Humans, IEEE Transactions on, 2010. **40**(1): p. 185-197.
2. Galar, M., et al., *A Review on Ensembles for the Class Imbalance Problem: Bagging-, Boosting-, and Hybrid-Based Approaches.* Systems, Man, and Cybernetics, Part C: Applications and Reviews, IEEE Transactions on, 2012. **42**(4): p. 463--484.
3. Breiman, L., *Random forests.* Machine learning, 2001. **45**(1): p. 5-32.
4. Ruschhaupt, M., et al., *A compendium to ensure computational reproducibility in high-dimensional classification tasks.* Statistical applications in genetics and molecular biology, 2004. **3**: p. Article37.
5. Bradley, A.P., *The use of the area under the ROC curve in the evaluation of machine learning algorithms.* Pattern recognition, 1997. **30**(7): p. 1145-1159.

6. Hanley, J.A. and B.J. McNeil, *A method of comparing the areas under receiver operating characteristic curves derived from the same cases.* Radiology, 1983. **148**(3): p. 839-843.
7. Team, R.D.C., *R: A language and environment for statistical computing*, 2010: Vienna, Austria.
8. Raghavan, V., P. Bollmann, and G.S. Jung, *A critical investigation of recall and precision as measures of retrieval system performance.* ACM Transactions on Information Systems (TOIS), 1989. **7**(3): p. 205-229.
9. Hollander, M. and D.A. Wolfe, *Nonparametric statistical methods.* 1999.
