# Supplementary material for: Prediction of protein interaction types based on sequence and network features
Source: BMC Syst Biol. 2013 Dec 13;7(Suppl 6):S5. doi: 10.1186/1752-0509-7-S6-S5 (PMC4029746; doi:10.1186/1752-0509-7-S6-S5)
Supplement: Additional file 2 — Benchmarking methods for both SP/ME classification and obligate/non-obligate classifications. [file 1752-0509-7-S6-S5-S2.PDF]

## Additional file 2

This file contains the bottom 40 ranked features for both SP/ME classification and obligate/non-obligate classifications. The features are ranked based on their Wilcoxon ranked-sum test P-value

Table S1 Ranking of the top 40 features for the obligate and non-obligate classes based on their Wilcoxon ranked sum test P-values. The numbers in the name column refer to EGDV values for orbits (see Table IV). For the number of disordered binding regions, fraction of disordered amino acids, and ELM both values for protein A and B are combined into one distribution which has two values for each interaction. The Wilcoxon

| Rank | Feature name | Mean obligate | Mean non-obligate | P-value | Rank | Feature name | Mean obligate | Mean non-obligate | P-value |
|---|---|---|---|---|---|---|---|---|---|
| 41 | 9 | 0.0112 | 0.00883 | 0.0053 | 61 | 40 | 0.0124 | 0.0107 | 1 |
| 42 | 37 | 0.0104 | 0.00701 | 0.0098 | 62 | 53 | 0.00665 | 0.00561 | 1 |
| 43 | 67 | 0.00194 | 0.00086 | 0.013 | 63 | 34 | 0.0123 | 0.0107 | 1 |
| 44 | 35 | 0.00705 | 0.00456 | 0.013 | 64 | 21 | 0.012 | 0.0105 | 1 |
| 45 | 28 | 0.0192 | 0.0135 | 0.023 | 65 | 38 | 0.0074 | 0.00666 | 1 |
| 46 | 63 | 0.00238 | 0.00118 | 0.028 | 66 | 27 | 0.0149 | 0.0133 | 1 |
| 47 | 19 | 0.0196 | 0.015 | 0.04 | 67 | 54 | 0.00514 | 0.00469 | 1 |
| 48 | APR | 0.864 | 0.778 | 0.04 | 68 | 58 | 0.0032 | 0.00369 | 1 |
| 49 | 66 | 0.00278 | 0.00149 | 0.04 | 69 | 22 | 0.0468 | 0.0441 | 1 |
| 50 | 29 | 0.0126 | 0.0152 | 0.047 | 70 | 42 | 0.00853 | 0.00793 | 1 |
| 51 | 14 | 0.0389 | 0.0446 | 0.049 | 71 | 48 | 0.00473 | 0.00525 | 1 |
| 52 | 62 | 0.00329 | 0.00178 | 0.072 | 72 | 56 | 0.00281 | 0.00283 | 1 |
| 53 | 61 | 0.00406 | 0.00273 | 0.076 | 73 | 64 | 0.00164 | 0.00155 | 1 |
| 54 | 59 | 0.00317 | 0.0016 | 0.16 | 74 | 3 | 0.0541 | 0.0504 | 1 |
| 55 | 55 | 0.00447 | 0.00309 | 0.18 | 75 | 13 | 0.087 | 0.0791 | 1 |
| 56 | 65 | 0.00218 | 0.00133 | 0.26 | 76 | 47 | 0.00414 | 0.00414 | 1 |
| 57 | 1 | 0.0313 | 0.0231 | 1 | 77 | 36 | 0.0103 | 0.0098 | 1 |
| 58 | 46 | 0.00907 | 0.00727 | 1 | 78 | 60 | 0.00305 | 0.00296 | 1 |
| 59 | 10 | 0.00553 | 0.00472 | 1 | 79 | 15 | 0.082 | 0.0785 | 1 |
| 60 | 16 | 0.0564 | 0.0591 | 1 | | | | | |

P-value is then calculated for this distribution.

Table S2 Ranking of the top 40 features for the simultaneously possible and mutually exclusive classes based on their Wilcoxon ranked sum test P-values. The numbers in the name column refer to EGDV values for orbits (see Table IV). For the number of disordered binding regions, fraction of disordered amino acids, and ELM both values for protein A and B are combined into one distribution which has two values for each interaction. The Wilcoxon P-value is then calculated for this distribution.

| Rank | Feature name | Mean SP | Mean ME | P value | Rank | Feature name | Mean SP | Mean ME | P value |
|---|---|---|---|---|---|---|---|---|---|
| 41 | 63 | 0.00113 | 0.00199 | 6e-16 | 61 | 27 | 0.0129 | 0.016 | 8.4e-05 |
| 42 | 51 | 0.00355 | 0.00557 | 1.1e-13 | 62 | MF | 0.75 | 0.715 | 0.00024 |
| 43 | 12 | 0.00135 | 0.00187 | 1.2e-13 | 63 | 29 | 0.0147 | 0.0168 | 0.00052 |
| 44 | 11 | 0.00194 | 0.00259 | 2.9e-13 | 64 | 28 | 0.0162 | 0.0167 | 0.00076 |
| 45 | 49 | 0.00134 | 0.00213 | 3.1e-12 | 65 | MaxDisLen | 35.4 | 33.1 | 0.0049 |
| 46 | 35 | 0.00488 | 0.0071 | 9.7e-12 | 66 | 30 | 0.0287 | 0.0268 | 0.0092 |
| 47 | 52 | 0.0046 | 0.00609 | 9.7e-12 | 67 | 4 | 0.022 | 0.0215 | 0.068 |
| 48 | CC | 0.793 | 0.764 | 1.4e-11 | 68 | DisRegions | 5.55 | 5.28 | 0.07 |
| 49 | 31 | 0.02 | 0.0243 | 1.6e-11 | 69 | 8 | 0.0104 | 0.0101 | 0.4 |
| 50 | 26 | 0.0227 | 0.0273 | 2.4e-11 | 70 | Betweeness | 3476 | 3916 | 1 |
| 51 | 68 | 0.00055 | 0.001 | 4e-11 | 71 | 2 | 0.00662 | 0.00315 | 1 |
| 52 | 39 | 0.0165 | 0.0196 | 4.6e-11 | 72 | 19 | 0.0163 | 0.0182 | 1 |
| 53 | MeanSim | 0.714 | 0.677 | 7.3e-11 | 73 | ELM | 166 | 140 | 1 |
| 54 | APR | 0.825 | 0.742 | 1e-09 | 74 | 24 | 0.0178 | 0.0178 | 1 |
| 55 | 37 | 0.00783 | 0.00903 | 7.3e-09 | 75 | 20 | 0.0339 | 0.0344 | 1 |
| 56 | 41 | 0.00584 | 0.00733 | 1.2e-08 | 76 | 25 | 0.0201 | 0.0175 | 1 |
| 57 | FracDisAS | 0.116 | 0.136 | 3.6e-08 | 77 | 9 | 0.00931 | 0.0106 | 1 |
| 58 | 5 | 0.0321 | 0.0298 | 5.9e-08 | 78 | 17 | 0.0315 | 0.0325 | 1 |
| 59 | 21 | 0.0104 | 0.0137 | 1.9e-07 | 79 | 18 | 0.0372 | 0.038 | 1 |
| 60 | BP | 0.599 | 0.554 | 7.9e-07 | | | | | |
