# Supplementary material for: Prediction of protein interaction types based on sequence and network features
Source: BMC Syst Biol. 2013 Dec 13;7(Suppl 6):S5. doi: 10.1186/1752-0509-7-S6-S5 (PMC4029746; doi:10.1186/1752-0509-7-S6-S5)
Supplement: Additional file 3 — Benchmarking results for both SP/ME classification and obligate/non-obligate classifications. [file 1752-0509-7-S6-S5-S3.PDF]

Additional file 3

Class balancing

Both datasets used in this work for training the classifiers are characterized by strong class imbalance. Specifically, in the obligate/non-obligate dataset the fraction of obligate interactions is 0.20 while in the SP/ME dataset the fraction of SP interactions is 0.36. We plotted precision and recall values for each interaction class for various fractions of the minority class (obligate, SP) in the training set (Figure S1), since we were interested in how the class balance affects the classifier performance. With increasing fraction of the minority class in the training data the classifier precision for the minority class declines, but its recall for this class increases; the opposite tendency was observed for the majority class (SP, non-obligate). We therefore sought to identify the optimal values of the two parameters of the RUSBoost method – the fraction of the minority class in the training set and the number of iterations – and to evaluate their influence on the classification results.

Overall, the random forest classifier was very robust with respect to different fractions of the minority class (Figure S2). Only when minority class instances constituted less than 10% or more then 90% of the data a severe effect on the auROC could be observed. Thus, according to auROC analysis, any value between 0.10 and 0.90 is acceptable. As for the F–measure (Figure S3), the obligate/non-obligate classifier was stable for fractions of obligate interactions between 0.40 and 0.60 while for the SP/ME classification F-measure values peaked between SP fractions of 0.30 and 0.40. As for the overall performance of the classifier (Figure S3, green line) we observed that for obligate/non-obligate classification the F-measure peaks at a value of 0.811 for the class ratio of 0.37, and for SP/ME classification the highest F-measure value of 0.829 was reached with the ratio of 0.31. As an additional test we trained one random forest classifier with 100

trees, with all features selected and no class balancing, and obtained F-measure values of 0.804 for obligate/non-obligate interactions and 0.811 for SP/ME interactions, respectively, evaluated in a ten–fold cross–validation experiment. This implies that utilization of these class ratios for the RUSBoost method can slightly improve the F-measure of both classifiers. Thus in this work, for all further analyses, we decided to use the ratio of 0.37 for the obligate/non-obligate classification and 0.31 for the SP/ME classification. Even though we only achieved a small improvement through class balancing, we were able to demonstrate the robustness of our classifier for a broad range of class ratios.

With regard to the number of RUSBoost iterations no significant improvement could be achieved for more than five iterations as judged by the auROC values (Figure S4). We therefore decided to use 10 iterations throughout this work to ensure optimal performance.

Predictor evaluation

In this subsection we describe performance evaluation of our random forest classifier for both obligate/non-obligate and SP/ME classification in comparison with the corresponding naïve classifiers, serving as baseline. The naïve classifier achieves an auROC value of 0.69 for the obligate/non-obligate classification using protein complex annotation information while for the SP/ME classification its performance is essentially random (auROC=0.54). Table S3 shows performance measures for each class in a 10–fold cross–validation. We observed higher precision, recall, and F-measure values for the majority classes (non-obligate, ME), than for the minority classes (obligate, SP). The reason for this is the fact that these evaluation metrics are susceptible to the class

imbalance in the data set. This is also the reason why we obtained the lowest values for the SP classification, since only 21% of the SP/ME dataset contains SP interactions. In terms of the overall performance, our classifier achieved the auROC values of 0.881 and 0.851 for the obligate/non-obligate and the SP/ME classification, respectively.

Table S4 shows the results of the ten–fold and nested–fold cross–validation for obligate/non-obligate classification, both with and without feature selection. For all feature selection methods we observed a minor decrease in performance compared to no feature selection. Information gain feature selection performed better than genetic and correlation-based feature selection, despite having the largest standard deviation. The results of the 10–fold and nested-fold cross–validation and holdout set analysis are quite similar, which demonstrates classifier robustness (Figure S5a,b and Figure S6a,b). There is also a significant improvement over the naïve classifier, justifying our use of additional features compared to the naïve version. However, there are noticeable jumps on the precision-recall curve for the holdout set (Figure S6a,b), presumably due to the small sample size.

We were primarily interested in the results of the correlation-based feature selection since it selected only 10% of all features (MF, BP, CC, meanSim, betweeness, FracDisASB, and MaxDisLen) at the cost of an essentially negligible decrease in performance. For obligate/non-obligate interactions, we observed a difference of 0.02 in auROC and F–measure between ten–fold cross–validation and nested–fold cross–validation. The deviation within the nested–fold cross–validation was merely around 0.01 (Table S4).

For SP/ME classification, correlation-based feature selection outperforms all other methods in 10–fold cross–validation, even without feature selection, while in nested–fold cross–validation it outperforms only all other feature selection methods (Table S5). The selected features are: 26, 32, 33, 43, 50, 57, 64, degree, elmA, elmB, BP, and

MaxDisLen. The fact that "functional similarity based on biological process GO terms" (BP, Table 4) was selected by the correlation based feature selection method, serves as evidence that BP has at least some importance for the classification. We observed only small deviations between ten–fold cross–validation and nested–fold cross–validation and within nested–fold cross–validation (Figure S5c,d and Figure S6c,d).

Cross-species evaluation

In this section we evaluate the performance of our classifier trained on data from one organism and then tested on data from another organism. The goal of cross species evaluation is to determine whether or not we can apply our classifier to species other than human, yeast, and *E. coli*. Classifiers trained and evaluated on data from the same organism have larger auROC values (Table S6, diagonal elements) than those obtained in all possible cross-species validation experiments (Table S6, off-diagonal elements). The only exception is constituted by the obligate/non-obligate classifier trained on human data and evaluated on *E. coli*, which has a somewhat larger auROC than the classifier trained and evaluated on *E. coli* data. This might be caused by the fact that the *E. coli* obligate/non-obligate dataset (60 interactions) is considerably smaller than the human obligate/non-obligate dataset (545 interactions). In general, we see that for obligate/non-obligate interactions classifiers trained on individual organism-specific datasets perform only marginally better on these native datasets (difference in auROC values between 0.01 and 0.05) than on data from organisms they have not been trained on. Interestingly, for SP/ME interactions the difference between organism-specific and cross species evaluation is slightly higher (between 0.05 and 0.1), but is still quite acceptable. This might have been caused by the fact that the obligate/non-obligate dataset was generated by the structure-based NOXclass classifier and is thus more homogenous. In conclusion these results suggests that both the obligate/non-obligate

and SP/ME classifiers can be applied to analyze interaction data from species other than the three organisms considered in this work, albeit with a slightly larger decline (up to 0.1) in auROC for the SP/ME classifier.

Table S3. Evaluation metrics for obligate, non-obligate, SP, ME classification for 10–fold cross–validation. auROC describes how well the classifier can distinguish both classes, hence there is only one value for each classifier (obligate/non-obligate, SP/ME).

	Obligate	Non-obligate	SP	ME
Precision	0.75	0.83	0.61	0.87
Recall	0.68	0.87	0.53	0.91
F-measure	0.71	0.85	0.56	0.88
auROC	0.881		0.851	

Table S4. Evaluation of feature selection methods for the obligate/non-obligate classification. The "Number of features" column refers to the average number of selected features in each fold. For nested–fold cross fold validation the standard deviation for each value is given with the "±" symbol. Performance measures (auROC and F-measure) reflect the overall performance of the classifier.

	10–fold cross–validation			Nested–fold cross validation		
Feature selection method	Number of features	F–measure	auROC	Number of features	F–measure	auROC
No feature selection	82	0.811	0.881	82	0.794±0.009	0.869±0.015
Information gain	20	0.803	0.877	20	0.804±0.017	0.871±0.019
Correlation	8.7	0.807	0.865	9.33	0.797±0.012	0.863±0.014
Genetic	38	0.808	0.880	35.6	0.793±0.017	0.869±0.015

Table S5. Evaluation of feature selection methods for SP/ME classification. The "Number of features" column refers to the average number of selected features in each fold. For nested–fold cross fold validation the standard deviation for each value is given with the "±" symbol. Performance measures (auROC and F-measure) reflect the overall performance of the classifier.

	10–fold cross–validation			Nested–fold cross validation		
Feature selection method	Number of features	F–measure	auROC	Number of features	F–measure	auROC
No feature selection	82	0.829	0.851	82	0.802±0.008	0.808±0.003
Information gain	20	0.783	0.791	20	0.769±0.004	0.769±0.011
Correlation	14.1	0.81	0.837	13.6	0.794±0.009	0.812±0.011
Genetic	41.7	0.837	0.816	37	0.795±0.013	0.811±0.011

Table S6. Cross species evaluation for SP/ME (left number) and obligate/non-obligate (right number) classification. For *E. coli* only one number for obligate/non-obligate classification is shown since no SP/ME data is available for this organism. Presented are auROC values of the classifiers trained with the data from the organisms shown in table rows and evaluated on species shown in table columns. Diagonal values (same species used for training and evaluation) were derived by 10–fold cross–validation. The off-diagonal elements show cross-species evaluation where the classifier was trained on the row species and evaluated on the column species.

	Human	Yeast	*E. coli*
Human	0.842/0.832	0.750/0.824	0.808
Yeast	0.768/0.718	0.814/0.766	0.731
E. coli	0.726	0.719	0.761

Figure S1. Precision and Recall values for different fractions of obligate (a), non-obligate (b), SP (c), and ME (d) interactions in the training set.

Figure S2. auROC of the classifier for different fractions of obligate (a) and SP (b) interactions in the training set.

Figure S3. F–measure of the classifier for different fractions of obligate (a) and SP (b) interactions in the training set.

Figure S4. auROC for different numbers of RUSBoost iterations.

Figure S5. ROC curves for obligate (a), non-obligate (b), SP (c), and ME (d) classification.

Figure S6. Precision/recall curves for obligate (a), non-obligate (b), SP (c), and ME (d) classification.

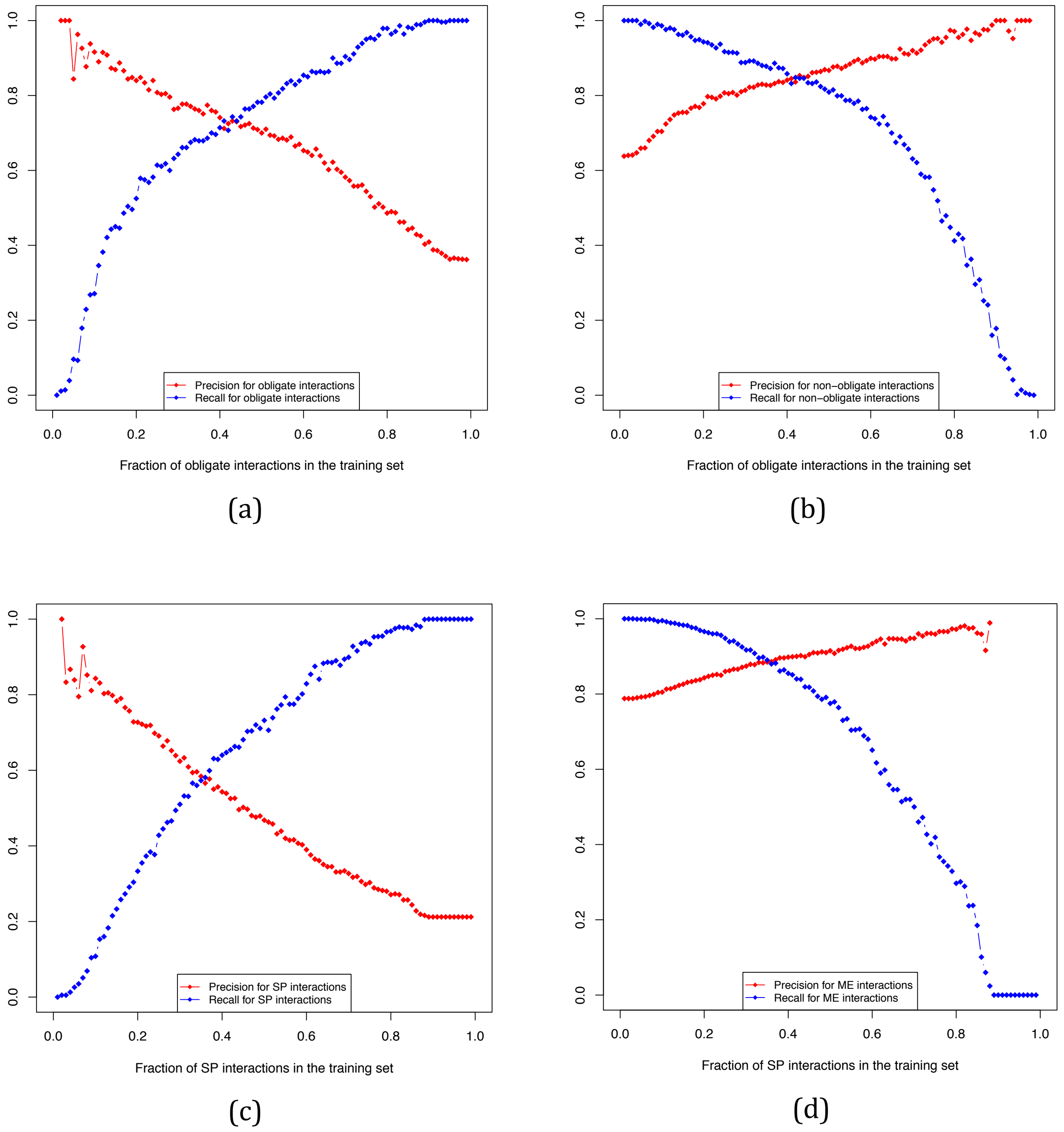
Precision for obligate interactions
Recall for obligate interactions
Fraction of obligate interactions in the training set
(a)
Precision for non–obligate interactions
Recall for non–obligate interactions
Fraction of obligate interactions in the training set
(b)
Precision for SP interactions
Recall for SP interactions
Fraction of SP interactions in the training set
(c)
Precision for ME interactions
Recall for ME interactions
Fraction of SP interactions in the training set
(d)
0.0
0.2
0.4
0.6
0.8
1.0

Figure S1

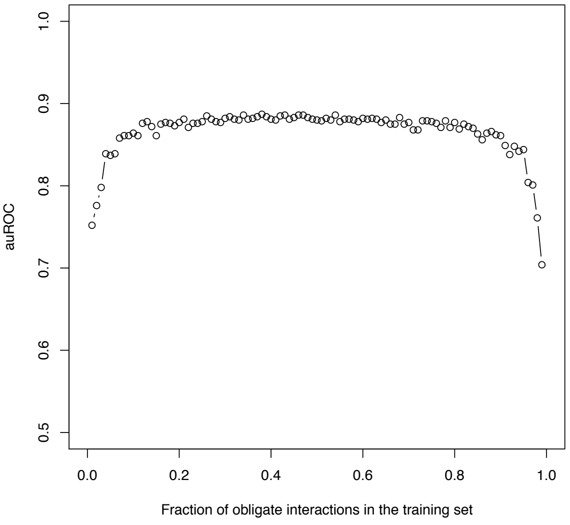

(a)

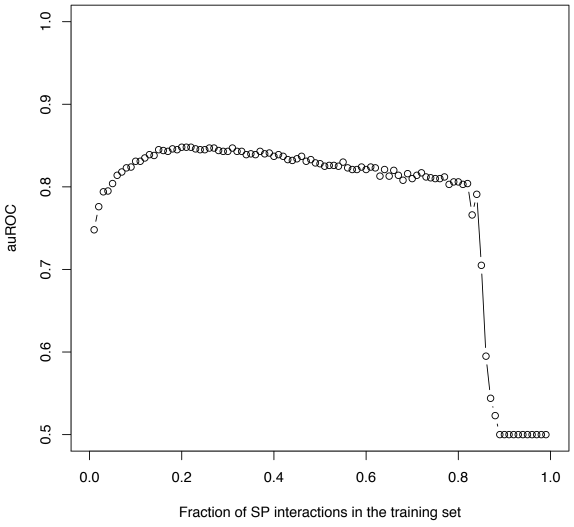

(b)

Figure S2

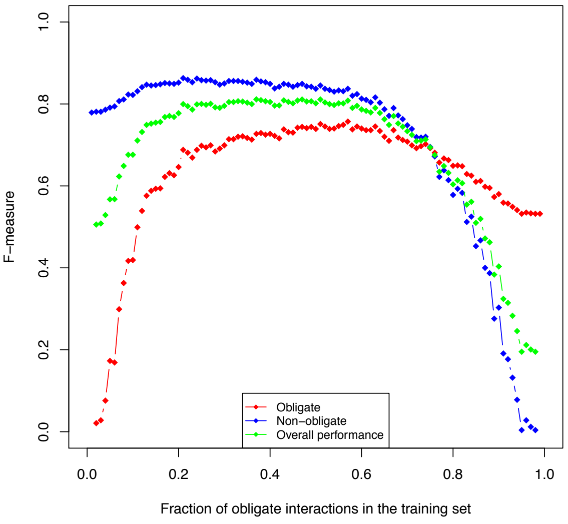

(a)

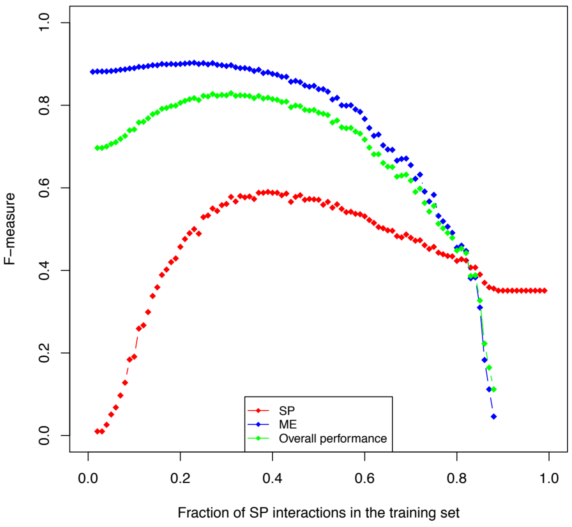

(b)

Figure S3

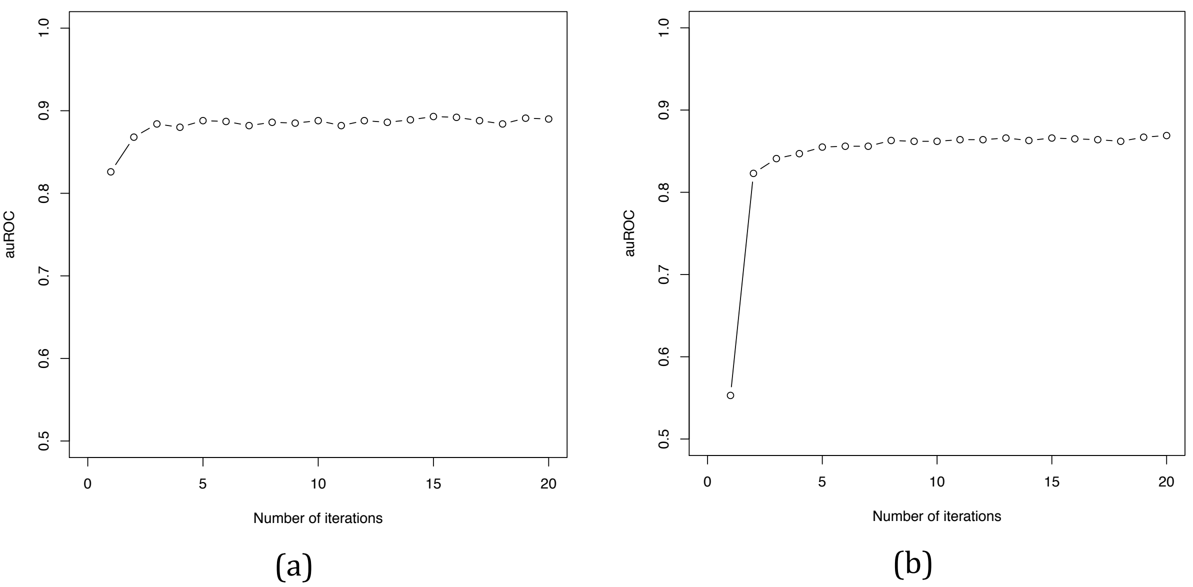
auROC
1.0
0.9
0.8
0.7
0.6
0.5
0
5
10
15
20
Number of iterations
(a)
auROC
1.0
0.9
0.8
0.7
0.6
0.5
0
5
10
15
20
Number of iterations
(b)

Figure S4

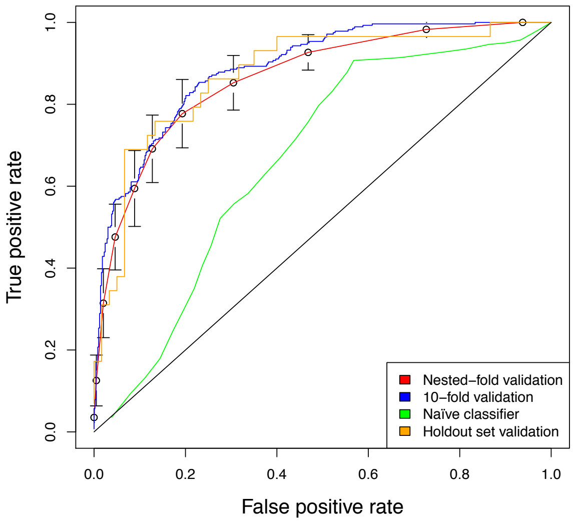

(a)

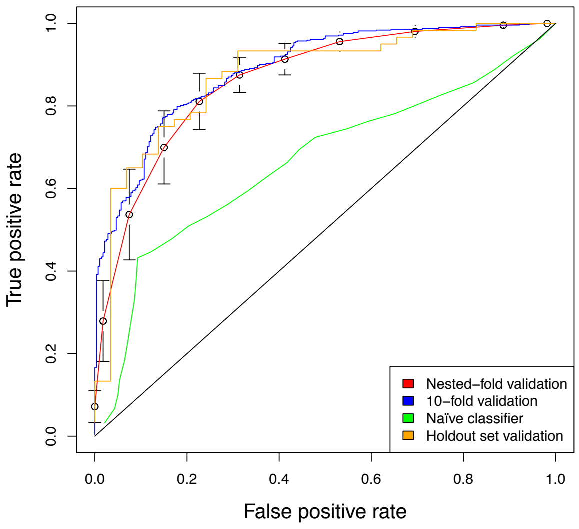

(b)

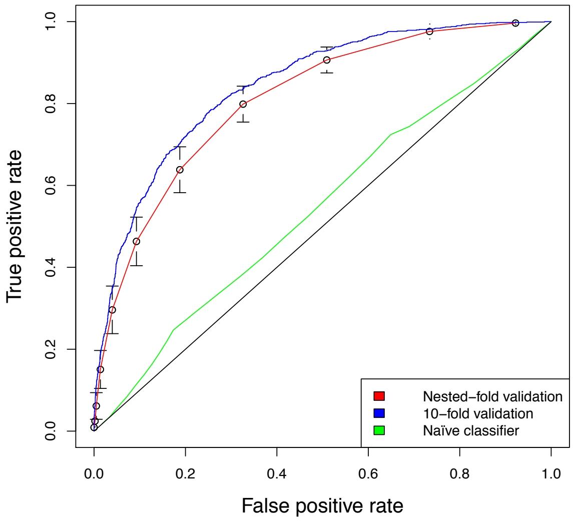

(c)

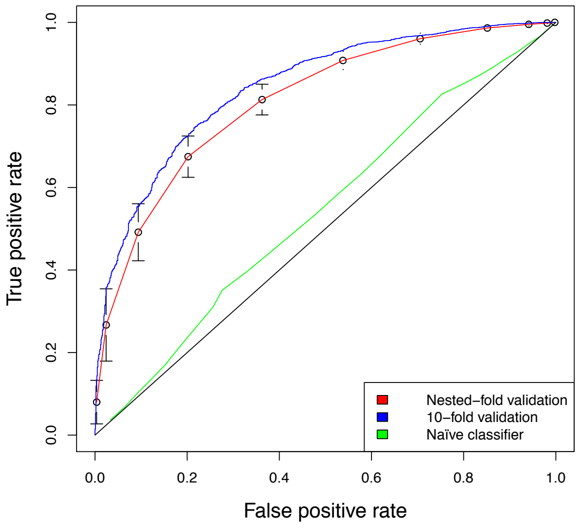

(d)

Figure S5

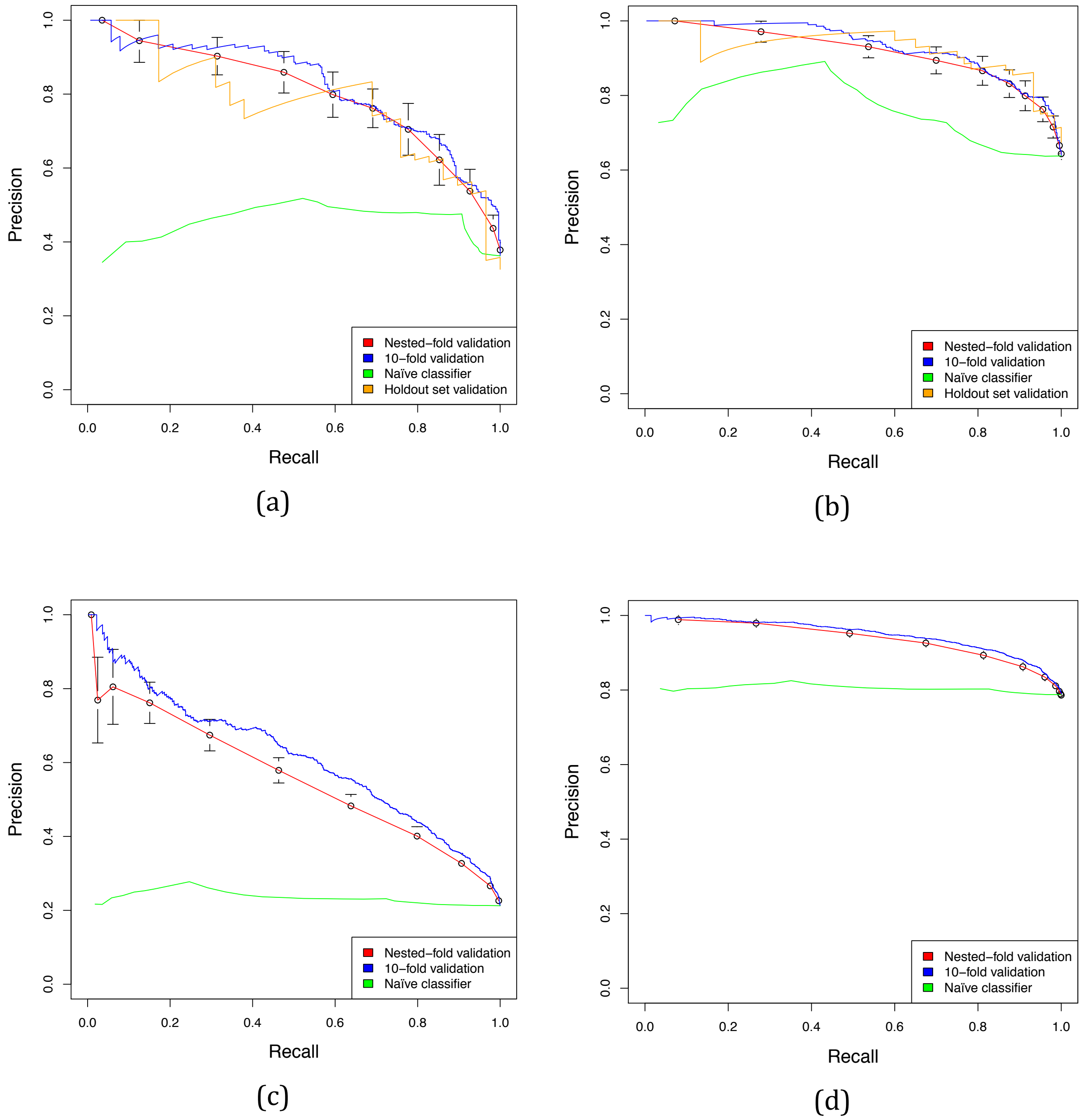

(a)
Precision
Recall
Nested–fold validation
10–fold validation
Naïve classifier
Holdout set validation
(b)
Precision
Recall
Nested–fold validation
10–fold validation
Naïve classifier
Holdout set validation
(c)
Precision
Recall
Nested–fold validation
10–fold validation
Naïve classifier
(d)
Precision
Recall
Nested–fold validation
10–fold validation
Naïve classifier

Figure S6
