# Supplementary material for: Prediction of protein interaction types based on sequence and network features
Source: BMC Syst Biol. 2013 Dec 13;7(Suppl 6):S5. doi: 10.1186/1752-0509-7-S6-S5 (PMC4029746; doi:10.1186/1752-0509-7-S6-S5)
Supplement: Additional file 4 — GO enrichment results. [file 1752-0509-7-S6-S5-S4.PDF]

Additional file 4

GO enrichment analysis

We applied GO enrichment analysis to explore the functional context of various types of interactions. As seen in Table S7 the overlap between different class combinations in terms of GO categories associated with them is very low, implying that each interaction type is intrinsic for a distinct set of cellular functions. The only deviation from this trend is the noticeable cross-talk between obligate/SP and obligate/ME interactions that share 52 GO terms, but only 8 of those actually describe molecular function while the remaining 44 shared GO terms refer to biological processes and cellular components. These two class combinations thus appear to share the same cellular location and to be involved in the same biological processes, yet to carry out distinctly different molecular functions. Table S8 shows a manually curated selection of the highest ranked (according to P-value) enriched GO terms. SP/obligate interactions are enriched in GO terms associated with nucleotide and nucleoside biosynthesis, their catabolism, as well as DNA replication and transcription. Proteins involved in these processes form stable multimeric complexes where they interact with their partners simultaneously. SP/non-obligate interactions frequently mediate cell-cell signaling, as exemplified in section 3.5 for biological vesicles. As for ME/obligate interactions, they are mostly associated with the GO terms describing complex subunit organization and lipid metabolic process and frequently occur in complexes with a ring shaped quaternary structure, such as the fatty acid synthase [1], the proteasome [2], and the U1 splicosome [3]. ME/non-obligate interactions are enriched in GO terms describing the regulation of various biological processes and seem to play a key role in signal transduction.

Table S7. Number of enriched GO terms for each class combination (diagonal line) and number of overlapping GO terms for each pair of class combinations (non–diagonal entries). Each cell contains counts of molecular function, cellular component, and biological process GO terms.

Obligate and SP	58/107/273			
Obligate and ME	8/21/23	23/57/55		
Non-obligate and SP	3/0/1	0/0/0	8/15/34	
Non-obligate and ME	0/2/1	1/4/0	0/1/9	28/33/115
	Obligate and SP	Obligate and ME	Non-obligate and SP	Non-obligate and ME

Table S8. Manual non-redundant selection of the first 20 to 25 highest ranked enriched GO terms for each interaction type.

	Enriched GO terms
SP and obligate	Nucleotide/nucleoside biosynthetic/catabolic processes
	DNA/RNA polymerase
	DNA replication
	RNA transcription
SP and non-obligate	Cell adhesion
	Cell communication
	Locomotion
	Cell junction assembly
	Cell recognition
	Cell-cell signaling
	Generation of a signal involved in cell-cell signaling
	Cell projection organization
	Cell junction organization
ME and obligate	Membrane lipid metabolic process
	Fatty acid elongation
	Lipid biosynthetic process
	Macromolecular complex subunit organization
	Ribonucleoprotein complex subunit organization
	Cellular macromolecular complex subunit organization
ME and non-obligate	Positive/negative regulation of biosynthetic process
	Positive/negative regulation of metabolic process
	Positive/negative regulation of cellular process

1. Jenni, S., et al., *Structure of fungal fatty acid synthase and implications for iterative substrate shuttling.* Science, 2007. **316**(5822): p. 254-261.
2. Whitby, F.G., et al., *Structural basis for the activation of 20S proteasomes by 11S regulators.* Nature, 2000. **408**(6808): p. 115-120.
3. Kambach, C., et al., *Crystal structures of two Sm protein complexes and their implications for the assembly of the spliceosomal snRNPs.* Cell, 1999. **96**(3): p. 375-387.
